# ATF3 coordinates the survival and proliferation of cardiac macrophages and protects against ischemia–reperfusion injury

**DOI:** 10.1038/s44161-023-00392-x

**Published:** 2024-01-04

**Authors:** Yihui Shao, Yang Li, Yan Liu, Shuolin Zhu, Jianing Wu, Ke Ma, Guoqi Li, Shan Huang, Haichu Wen, Congcong Zhang, Xin-liang Ma, Ping Li, Jie Du, Yulin Li

**Affiliations:** 1 Beijing Anzhen Hospital of Capital Medical University and Beijing Institute of Heart Lung and Blood Vessel Diseases, Beijing, China; 2 Department of Emergency Medicine, Thomas Jefferson University, Philadelphia, PA USA

**Keywords:** Acute coronary syndromes, Immunology

## Abstract

Cardiac resident MerTK^+^ macrophages exert multiple protective roles after ischemic injury; however, the mechanisms regulating their fate are not fully understood. In the present study, we show that the GAS6-inducible transcription factor, activating transcription factor 3 (ATF3), prevents apoptosis of MerTK^+^ macrophages after ischemia–reperfusion (IR) injury by repressing the transcription of multiple genes involved in type I interferon expression (*Ifih1* and *Ifnb1*) and apoptosis (*Apaf1*). Mice lacking ATF3 in cardiac macrophages or myeloid cells showed excessive loss of MerTK^+^ cardiac macrophages, poor angiogenesis and worse heart dysfunction after IR, which were rescued by the transfer of MerTK^+^ cardiac macrophages. GAS6 administration improved cardiac repair in an ATF3-dependent manner. Finally, we showed a negative association of GAS6 and ATF3 expression with the risk of major adverse cardiac events in patients with ischemic heart disease. These results indicate that the GAS6–ATF3 axis has a protective role against IR injury by regulating MerTK^+^ cardiac macrophage survival and/or proliferation.

## Abstract (web summary)

Shao, Li, Liu et al. identify the GAS6–ATF3 axis as a central regulator of survival and proliferation of protective resident MerTK^+^ cardiac macrophages and show that GAS6 administration improves cardiac repair after ischemic–reperfusion injury in mice in an ATF3-dependent manner.

## Main

Myocardial infarction (MI) is the leading cause of morbidity and mortality worldwide^1^. Reperfusion therapy has dramatically reduced the acute mortality associated with MI. Although reperfusion is essential for myocardial salvage, it triggers cardiomyocyte death and a sterile immune response that results in further myocardial injury called IR injury. To date, pharmacological interventions have not been developed for IR injury, so it remains an unsolved clinical complication.

Accumulating evidence demonstrates that this immune response involves two equally important phases during IR as follows: the proinflammatory phase and the reparatory phase^2,3^. The balance between the two phases is crucial for efficient cardiac function recovery and improved prognosis. IR injury is characterized by the extensive accumulation of immune cells. Macrophages are one of the most abundant immune cells and represent a highly heterogeneous population. Infiltrating CCR2^+^ monocytes/macrophages promote myocardial inflammation, adverse remodeling and cardiac dysfunction after MI^4^. In contrast, cardiac macrophage subsets that lack CCR2 expression but coexpress myeloid–epithelial–reproductive receptor tyrosine kinase (MerTK) exert multiple protective effects on cardiac physiological and pathological states^5,6^. The primary function of MerTK^+^ cardiac macrophages is to eliminate dying cells or unwanted material by efferocytosis. Depletion of cardiac macrophages results in the accumulation of anomalous mitochondria in cardiomyocytes and activation of the inflammasome at a steady state^7^. During MI or IR, cardiac macrophages engulf dead cells via MerTK, thereby preventing inappropriate myocardial necrosis. MerTK depletion or MerTK cleavage increases the accumulation of apoptotic cells and cardiac dysfunction^8,9^. In addition, MerTK^+^ cardiac macrophages facilitate electrical conduction^10^, promote coronary development and angiogenesis^11^, inhibit monocyte recruitment^4^, promote postnatal cardiomyocyte proliferation^12^ and prevent cardiac remodeling^13^. Furthermore, MerTK^+^ cardiac macrophages display pro-reparative properties in human heart failure^14^. As such, strategies that target MerTK^+^ cardiac macrophages hold promise for the amelioration of ischemic heart disease.

For effective protection to take place, MerTK^+^ cardiac macrophage viability must be maintained; however, recent reports from independent groups have shown that the abundance of cardiac macrophages is remarkably reduced after MI^6^. Beyond these observations, a more detailed understanding of cardiac macrophage loss through cell death and other processes is lacking. Although studies have revealed that cardiac macrophages are exclusively replenished by local proliferation in both humans and mice^6,14^, how cell-intrinsic molecular mechanisms influence the survival and/or proliferation of MerTK^+^ cardiac macrophages after IR remains unclear. Understanding the cues that drive the fate of MerTK^+^ cardiac macrophages is critical for the development of therapeutic strategies to enhance MerTK^+^ cardiac macrophage populations.

In the present study, we used an unbiased approach to investigate the regulatory mechanism of MerTK^+^ cardiac macrophage fate in response to IR. Single-cell RNA sequencing (scRNA-seq) and functional studies revealed that MerTK^+^ cardiac macrophages exhibit enhanced death. The findings revealed that GAS6-inducible ATF3 acts as a central regulator of the survival and/or proliferation of MerTK^+^ cardiac macrophages after IR. GAS6 administration improves cardiac repair and functions in an ATF3-dependent manner. Finally, we confirmed the association between plasma GAS6 levels and cardiac repair and function in patients with myocardial ischemia. Taken together, our findings suggest that the GAS6–ATF3 axis regulates MerTK^+^ cardiac macrophage fate and may serve as a therapeutic strategy for IR injury.

## Results

### IR triggers a reduction in MerTK^+^ cardiac macrophages

**Fig. 1:**
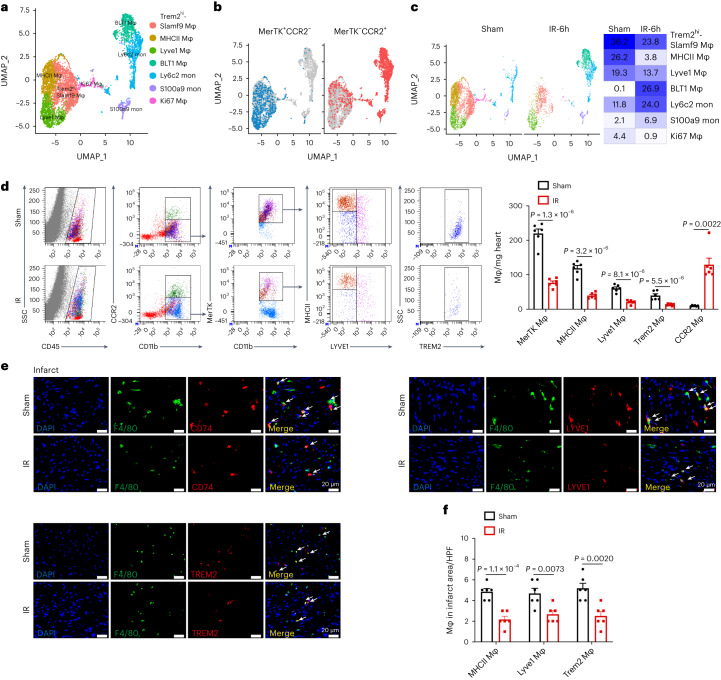
Myocardial IR induces a reduction in MerTK^+^ cardiac macrophage proportions. **a**, UMAP of seven macrophage (Mφ)/monocyte (mon) clusters identified via scRNA-seq analysis. **b**, UMAP visualization of MerTK^+^CCR2^−^ and MerTK^−^CCR2^+^ macrophages. **c**, Split UMAP of macrophage/monocyte clusters (left) and percentage of seven clusters (right) in the heart between sham operation and IR. **d**, Representative flow cytometry plots (left) and quantification (right) of MerTK^+^CCR2^−^ macrophages and MerTK^−^CCR2^+^ macrophages under sham operation and IR (*n* = 6 mice per group). **e**,**f**, Quantification (**e**) and colocalization (**f**) of IF signals of F4/80 and LYVE1, TREM2 and CD74 in cardiac infarct regions of the sham and IR (*n* = 6 mice per group). Scale bars, 20 μm. Statistical significance was evaluated using two-tailed, unpaired Student’s *t*-test (**d**: MerTK macrophage (Mφ), MHCII Mφ, Lyve1 Mφ, Trem2 Mφ; and **f**) or unpaired Mann–Whitney *U*-test (**d**: CCR2 Mφ). All data are presented as mean ± s.e.m. HPF, high-power field. Source data

**Extended Data Fig. 1:**
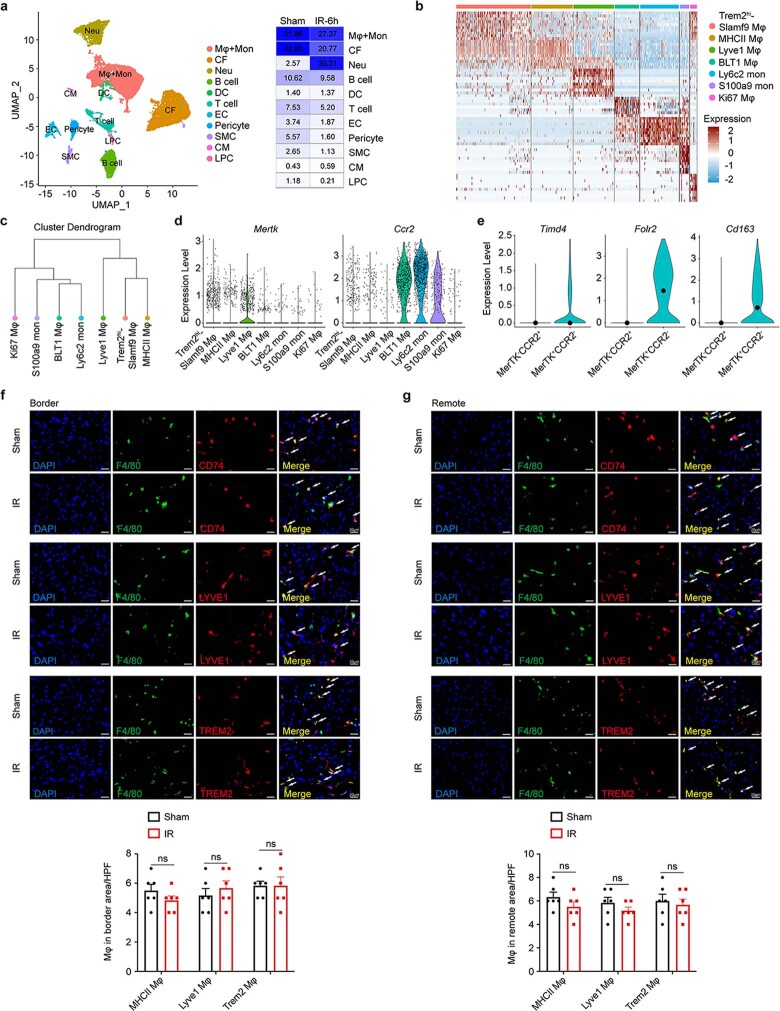
Identification of macrophage/monocyte subpopulations. **a**. Uniform manifold approximation and projection (UMAP) of single-cell RNA-sequencing (scRNA-seq) revealed 11 unique clusters (left) and the percentage of cells per cell type (right) in the heart between sham and IR. Mφ, macrophage; Mon, monocyte; CF, cardiac fibroblast; Neu, neutrophil; DC, dendritic cell; EC, endothelial cell; SMC, smooth muscle cell; CM, cardiomyocyte; LPC, lymphoid progenitor cell. **b**. Heatmap of the top ten differentially expressed genes (DEGs) in seven clusters of macrophages/monocytes. **c**. Hierarchical clustering of macrophages and monocytes. **d**. Violin plots representing log-normalized expression of *Mertk* and *Ccr2* in the seven macrophage/monocyte clusters. **e**. Violin plots representing the log-normalized expression of *Timd4*, *Folr2*, and *Cd163* in MerTK^+^CCR2^-^ macrophages and MerTK^−^CCR2^+^ macrophages. **f**, **g**. Colocalization (up) and quantification (down) of immunofluorescence (IF) signals of F4/80 and LYVE1, TREM2, and CD74 in cardiac border (**f**) and remote (**g**) regions of the sham and IR (n = 6 mice per group). Scale bar, 20 μm. HPF, high-power field. Statistical significance was evaluated using two-tailed unpaired *t* tests (**f** and **g**). All data are presented as mean ± SEM. Source data

To better understand the composition and alterations of macrophages during the early phase in response to IR, we performed scRNA-seq of heart tissues from sham and IR-operated mice 6 h after surgery using the 10× Genomics platform. After quality control filtering and deconvolution of the barcodes, we identified 18,726 unique cells, including 10,842 cells from sham samples and 7,884 cells from IR samples. Uniform manifold approximation and projection (UMAP) dimensionality reduction analyses identified 11 major populations and revealed that macrophages and monocytes are the most abundant and diverse cell populations in the leukocytes in heart tissues of sham or IR mice (Fig. 1a and Extended Data Fig. 1a,b). Hierarchical clustering analysis classified the macrophages and monocytes into seven populations (Extended Data Fig. 1c). We identified and recruited the cardiac macrophage/monocyte populations based on the expression of MerTK and CCR2. Collectively, the MerTK^+^CCR2^−^ population consisted of Trem2^+^, MHCII^+^ and Lyve1^+^ clusters, and the MerTK^−^CCR2^+^ population of BLT1^+^, Ki67^+^, Ly6c2^+^ and S100a9^+^ clusters (Fig. 1b and Extended Data Fig. 1d). MerTK^+^CCR2^−^ cardiac macrophages were characterized by high expression levels of *Timd4*, *Cd163* and *Flor2* (Extended Data Fig. 1e). A decrease in the proportions of MerTK^+^ cardiac macrophages (81.7% versus 41.3%) and an increase in those of CCR2^+^ macrophages (18.3% versus 58.7%) occurred in response to IR (Fig. 1c). We verified that the proportion of MerTK^+^CCR2^−^ macrophages, including the Trem2^+^, MHCII^+^ and Lyve1^+^ clusters, substantially decreased in the heart 6 h after IR (Fig. 1d). Immunostaining showed the decreased LYVE1^+^, TREM2^+^ and CD74^+^ expression density among F4/80^+^ macrophages in the heart after IR. These changes were predominantly observed in the IR area and not in the border or remote areas (Fig. 1e,f and Extended Data Fig. 1f,g). These data suggested that IR caused a profound decrease in MerTK^+^ cardiac macrophage populations.

### ATF3 regulates fate of MerTK^+^ cardiac macrophages during IR

**Fig. 2:**
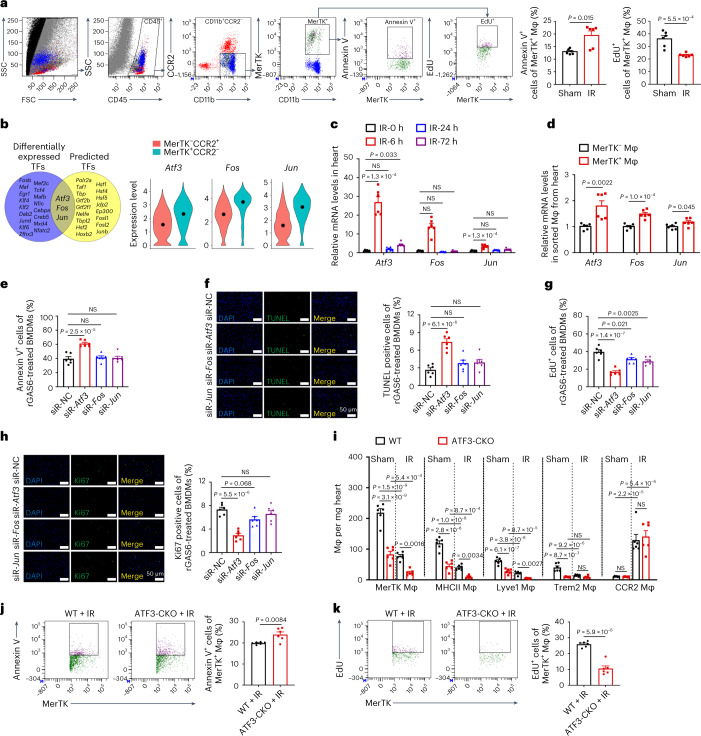
ATF3 as a key determinant for MerTK^+^ resident macrophage survival and/or proliferation in response to IR. **a**, Flow cytometry gating strategy and quantification of Annexin V^+^ and EdU^+^ cells in MerTK^+^ macrophages in the heart of the sham operation and IR groups (*n* = 6 mice per group). **b**, Overlap between differentially expressed TFs and RCisTarget-predicted TFs (left). Violin plots of normalized scRNA expression profiles of the three TFs in two types of macrophages (right). **c**, Relative mRNA expression of *Atf3*, *Jun* and *Fos* in the heart tissues at different time points after IR (*n* = 6 mice per group). **d**, Relative mRNA expression of *Atf3*, *Jun* and *Fos* in MerTK^+^ and MerTK^−^ macrophages sorted from the heart 6 h after IR (*n* = 6 mice per group). **e**, Flow cytometry analysis of Annexin V^+^ apoptotic cells in rGAS6-treated BMDMs transfected with the respective siRNAs (*n* = 6 biologically independent samples per group). **f**, Representative images (left) and quantification (right) of TUNEL-stained cells in rGAS6-treated BMDMs transfected with the respective siRNAs (*n* = 6 biologically independent samples per group). Scale bars, 50 μm. **g**, Flow cytometry analysis of EdU incorporation into rGAS6-treated BMDMs transfected with the respective siRNAs (*n* = 6 biologically independent samples per group). **h**, Representative images (left) and quantification (right) of Ki67^+^ cells in rGAS6-treated BMDMs transfected with the respective siRNAs (*n* = 6 biologically independent samples per group). Scale bars, 50 μm. **i**, Quantification of MerTK^+^, Trem2^+^, Lyve1^+^, MHCII^+^ and CCR2^+^ macrophages from the hearts of the two genotypes 6 h after sham operation and IR (*n* = 6 mice per group). **j**,**k**, Flow cytometry analysis of Annexin V^+^MerTK^+^ (**j**) and EdU^+^MerTK^+^ (**k**) macrophages in the heart of mice with two genotypes 6 h after IR (*n* = 6 mice per group). Statistical significance was evaluated via two-tailed, unpaired Student’s *t*-test (**a**, EdU; **d**, Fos, Jun; **j** and **k**), unpaired Mann–Whitney *U*-test (**a**, Annexin V; **d**, *Atf3*), Kruskal–Wallis test followed by Dunn’s multiple-comparison test (**c**), one-way ANOVA followed by Tukey’s multiple-comparison test (**e**–**h**) and two-way ANOVA followed by Tukey’s multiple-comparison test (**i**). All data are presented as mean ± s.e.m. Source data

We hypothesized that the massive loss of MerTK^+^ cardiac macrophages was the result of excessive apoptosis and insufficient proliferation during IR. Consistent with this hypothesis, after IR, mice harbored higher proportions of Annexin V^+^MerTK^+^ and lower proportions of EdU^+^MerTK^+^ cardiac macrophages compared with sham mice (Fig. 2a).

**Extended Data Fig. 2:**
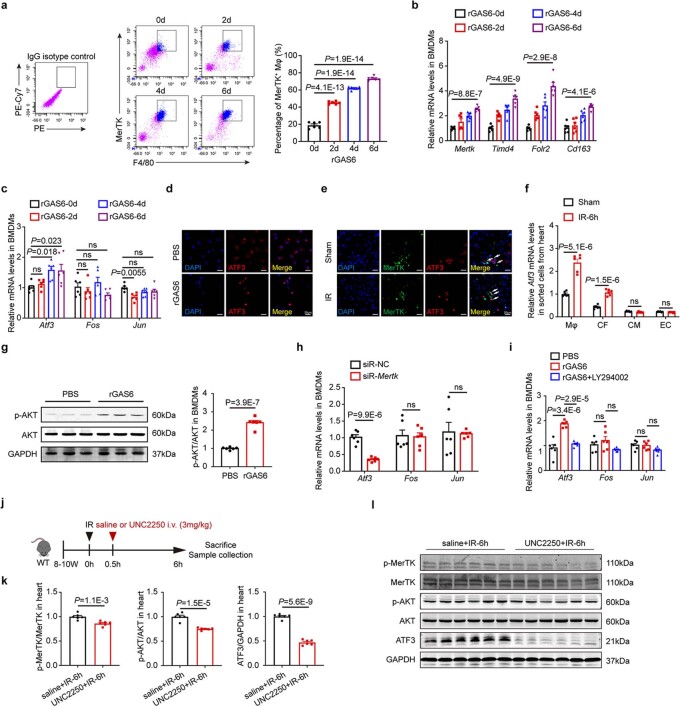
Role of GAS6-MerTK signaling in ATF3 induction of macrophage. **a**. Flow cytometry analysis and quantification of MerTK^+^ macrophages in bone marrow-derived macrophages (BMDMs) treated with rGAS6 at each time point (n = 6 per group). **b**. Relative mRNA expression of *Mertk*, *Timd4*, *Folr2*, and *Cd163* in the rGAS6-treated BMDMs at different time points (n = 6 biologically independent samples per group). **c**. Relative mRNA expression of *Atf3*, *Jun*, and *Fos* in the rGAS6-treated BMDMs at different time points (n = 6 biologically independent samples per group). **d**. Representative IF images of ATF3 in BMDMs treated with rGAS6 or PBS for 24 h. Scale bar, 20 μm. This experiment was repeated independently triple with similar results. **e**. Representative IF images of MerTK (green) and ATF3 (red) in the heart of the sham operation and IR 6 h post-MI. Scale bar, 20 μm. This experiment was repeated independently triple with similar results. **f**. Relative mRNA expression of *Atf3* in Mφ, CF, CM, and EC sorted from the heart of the sham and IR (n = 6 mice per group). **g**. Representative western blot analysis and quantification of the expression of phosphorylated- and total-AKT in BMDMs treated with rGAS6 for 1 h (n = 6 biologically independent samples per group). **h**. Relative mRNA expression of *Atf3*, *Jun*, and *Fos* in rGAS6-treated BMDMs transfected with scrambled control or *Mertk*-siRNA (n = 6 biologically independent samples per group). **i**. Relative mRNA expression of *Atf3*, *Jun*, and *Fos* in BMDMs pretreated with vehicle or LY294002 (a AKT inhibitor) (n = 6 biologically independent samples per group). **j**. Outline of UNC2250 (3 mg/kg) or saline treatment in WT mice subjected to IR surgery. **k**, **l**. Representative western blot analysis (**k**) and quantification (**l**) of the expression of phosphorylated- and total-MerTK, phosphorylated- and total-AKT, and ATF3 in the heart of IR mice treated with saline or UNC2250 (n = 6 mice per group). Statistical significance was evaluated using two-tailed unpaired *t* tests (**f**, **g**, **h**-*Atf3*, *Jun*, and **k**), unpaired Mann-Whitney *U* test (h-*Fos*), and one^−^way ANOVA analysis followed by Tukey’s multiple comparisons test (**a**–**c**, **i**). All data are presented as mean ± SEM. Source data

To elucidate the molecular determinants of MerTK^+^ cardiac macrophage survival and/or proliferation, we performed systematic analyses and functional experiments. We first identified differentially expressed transcription factors (TFs) (*P* < 0.05 and fold-change >1.5) between MerTK^+^ cardiac macrophages and MerTK^−^ macrophages. Subsequently, we predicted 20 TFs responsible for the signature genes of MerTK^+^ cardiac macrophages using the RCisTarget algorithm. *Atf3*, *Fos* and *Jun* were at the intersection of the two groups of TFs (Fig. 2b and Supplementary Table 1). Indeed, *Atf3*, *Fos* and *Jun* expressions were mainly increased in the early phase of IR and were higher in MerTK^+^ cardiac macrophages than in MerTK^−^ macrophages sorted from the heart after IR (Fig. 2c,d). Bone marrow-derived macrophages (BMDMs) were stimulated with recombinant GAS6 (rGAS6), the best-characterized ligand for MerTK, to obtain MerTK-like macrophages in vitro. These macrophages were characterized by gradually increased expression of *Mertk*, *Cd163*, *Timd4* and *Folr2* (Extended Data Fig. 2a,b). The rGAS6 increased *Atf3* expression in a time-dependent manner, but the elevation of *Fos* and *Jun* expression was not evident (Extended Data Fig. 2c). Nuclear translocation of ATF3 was observed in rGAS6-induced MerTK^+^ macrophages and MerTK^+^ macrophages in the heart after IR (Extended Data Fig. 2d,e). *Atf3* messenger RNA expression increased in both macrophages and cardiac fibroblasts sorted from the heart after IR, but this elevation is more pronounced in macrophages (Extended Data Fig. 2f).

Using small interfering (si)RNA-mediated knockdown, we tested which TFs were the key drivers of MerTK^+^ cardiac macrophage fate ex vivo. We found that knockdown of *Atf3*, but not *Fos* or *Jun*, increased the frequency of Annexin V^+^MerTK^+^ macrophages and terminal deoxynucleotidyl transferase dUTP nick-end labeling (TUNEL)-stained positive macrophages compared with the siRNA-negative control (NC)-transfected cells under HR conditions (Fig. 2e,f). Proliferation was decreased most precipitously in cultured MerTK^+^ macrophages transfected with siRNA–*Atf3*, as determined via the ethynyl deoxyuridine (EdU) assay and immunostaining for Ki67, a marker of proliferating cells (Fig. 2g,h). Considering that ATF3 is highly expressed in MerTK^+^ cardiac macrophages, we tested whether GAS6–MerTK signaling is responsible for their upregulation. Exposing macrophages to rGAS6 increased AKT phosphorylation downstream of MerTK (Extended Data Fig. 2g). The siRNA-mediated knockdown of MerTK or inhibition of AKT abolished the effects of rGAS6 on *Atf3* induction in vitro but did not affect *Fos* and *Jun* expression (Extended Data Fig. 2h,i). We tested whether MerTK signaling leads to ATF3 upregulation in vivo. WT mice were intravenously administered saline or UNC2250 (a selective inhibitor for MerTK)^15,16^. UNC2250 decreased phosphorylation of MerTK and downstream signaling proteins of AKT in the heart, which demonstrated the inhibition effect. The levels of ATF3 protein were reduced in the heart after UNC2250 administration (Extended Data Fig. 2j–l). The consistency between the in vitro and in vivo data confirmed the role of MerTK signaling on ATF3 induction.

**Extended Data Fig. 3:**
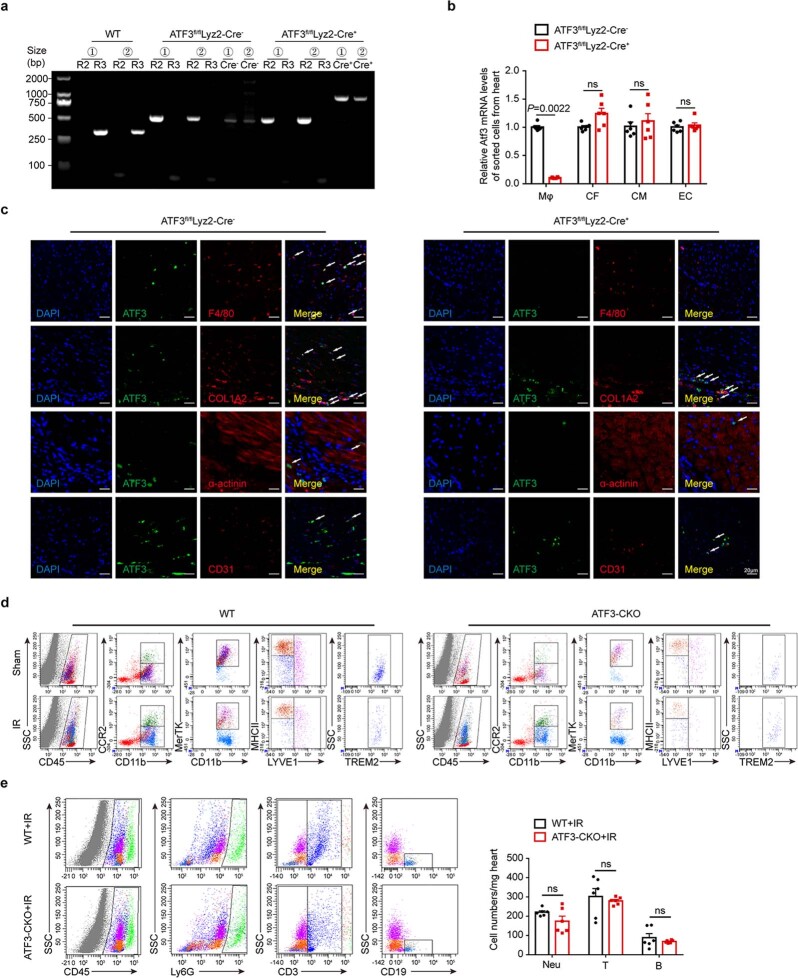
Flow cytometry analysis of macrophages and other immune cells. **a**. Representative agarose gel images of PCR results using DNA extracted from the tail as a template in ATF3-CKO and WT littermate control mice. **b**. Relative mRNA expression of Atf3 in Mφ, CF, CM, and EC sorted from ATF3-CKO and WT mice (n = 6 mice per group). **c**. Representative IF images of ATF3 (green) and F4/80, Col1a2, CD31, and α-actinin (red) in the heart of ATF3-CKO and WT mice. Scale bar, 20 μm. This experiment was repeated independently triple with similar results. **d**. Representative flow cytometry plots of MerTK^+^ macrophages and the three clusters in ATF3-CKO and WT mice under sham and IR. **e**. Representative dot plots and quantification of neutrophils, T cells, and B cells in the heart of ATF3-CKO and WT mice post-IR (n = 6 mice per group). Statistical significance was evaluated using two-tailed unpaired *t*-test (b, e). All data are presented as mean ± SEM. Source data

Next, we generated conditional, myeloid-specific, ATF3 knockout mice (ATF3^fl/fl^Lyz2-cre^+^, ATF3-CKO) to examine the role of ATF3 in macrophages in vivo. Reverse transcription–quantitative PCR (RT–qPCR) and immunofluorescence (IF) staining showed that ATF3 was specifically deleted in macrophages, but not in cardiac fibroblasts, endothelial cells (ECs).or cardiomyocytes (Extended Data Fig. 3a–c). ATF3^fl/fl^Lyz2-cre^−^ mice served as wild-type (WT) controls. Under steady-state conditions, ATF3 deletion led to a reduction in the frequency of MerTK^+^ cardiac macrophages; when mice received IR, we consistently found a notably decreased number of MerTK^+^ cardiac macrophages and three clusters in ATF3-CKO hearts compared with WT hearts (Fig. 2i and Extended Data Fig. 3d). Other investigated immune cells (neutrophils, B cells and T cells) had comparable frequencies in ATF3-CKO and WT hearts (Extended Data Fig. 3e). We observed an increase in the frequency of Annexin V^+^MerTK^+^ and a decrease in that of EdU^+^MerTK^+^ cardiac macrophages in ATF3-CKO mice compared with WT mice after IR (Fig. 2j,k). Together, these data suggested that GAS6–MerTK signaling induced ATF3 in macrophages and that ATF3 controlled the survival and/or proliferation of MerTK^+^ cardiac macrophages.

### ATF3 controls MerTK^+^ macrophages by inhibiting type I IFN

**Fig. 3:**
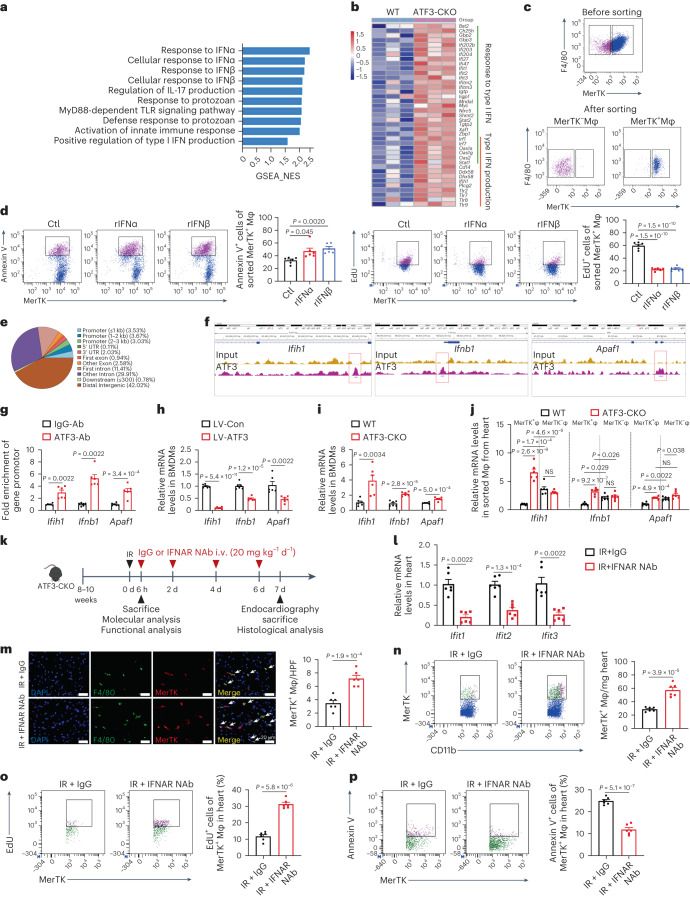
ATF3 controls MerTK^+^ macrophage fate via inhibiting type I IFN. **a**, GSEA comparing rGAS6-stimulated BMDMs from ATF3-CKO and WT mice (*n* = 3 mice). NES, normalized enrichment score. **b**, Heatmap of DEGs in type I IFN signals. **c**,**d**, Flow cytometry analysis of Annexin V^+^ and EdU^+^ cells in sorted MerTK^+^ macrophages treated with rIFNα (**c**) or rIFNβ (**d**; *n* = 6 biologically independent samples). **e**, Pie chart of the percentage of peaks within each category bound by ATF3. **f**, Genome browser view of ChIP–seq tracks for ATF3 at the *Ifih1*, *Ifnb1* and *Apaf1* loci in BMDMs. **g**, ChIP–qPCR analysis of ATF3 binding to the *Ifih1*, *Ifnb1* and *Apaf1* promoter regions in BMDMs (*n* = 6 biologically independent samples). The signal was relative to the percentage input. **h**, Relative mRNA expression of *Ifih1*, *Ifnb1* and *Apaf1* in rGAS6-induced BMDMs infected with LV-Con or LV-ATF3 (*n* = 6 biologically independent samples). **i**, Relative mRNA expression of *Ifih1*, *Ifnb1* and *Apaf1* in rGAS6-stimulated BMDMs from ATF3-CKO and WT mice (*n* = 6 biologically independent samples). **j**, Relative mRNA expression of *Ifih1*, *Ifnb1* and *Apaf1* in MerTK^+^ and MerTK^−^ macrophages sorted from the heart of ATF3-CKO and WT mice 6 h after IR (*n* = 6 mice). **k**, Outline of IFNAR NAbs (20 mg kg^−1^ d^−1^) or IgG treatment in ATF3-CKO mice subjected to IR surgery. **l**, Relative mRNA expression of *Ifit1*, *Ifit2* and *Ifit*3 in the hearts of the two groups (*n* = 6 mice). **m**, Representative IF images and quantification of MerTK^+^ macrophages in the hearts of the two groups (*n* = 6 mice). Scale bars, 20 μm. **n**, Representative flow cytometry plots and quantification of MerTK^+^ macrophages in the hearts of the two groups (*n* = 6 mice). **o**,**p**, Flow cytometry analysis of EdU^+^ (**o**) and Annexin V^+^ (**p**) cells in MerTK^+^ macrophages in the hearts of the two groups (*n* = 6 mice). Statistical significance was evaluated using two-tailed, unpaired Student’s *t*-test (**g**, *Apaf1*; **h**, *Ifih1*, *Ifnb1*; **i** and **l**, *Ifit2*; and **m**–**p**), unpaired Mann–Whitney *U*-test (**g**, *Ifih1*, *Ifnb1*; **h,** *Apaf1*; **l,** *Ifit1*, *Ifit3*), one-way ANOVA followed by Tukey’s multiple-comparison test (**d**, EdU), Kruskal–Wallis test followed by Dunn’s multiple-comparison test (**d**, Annexin V) and two-way ANOVA followed by Tukey’s multiple-comparison test (**j**). All data are presented as mean ± s.e.m. Ctl, Control. Source data

**Extended Data Fig. 4:**
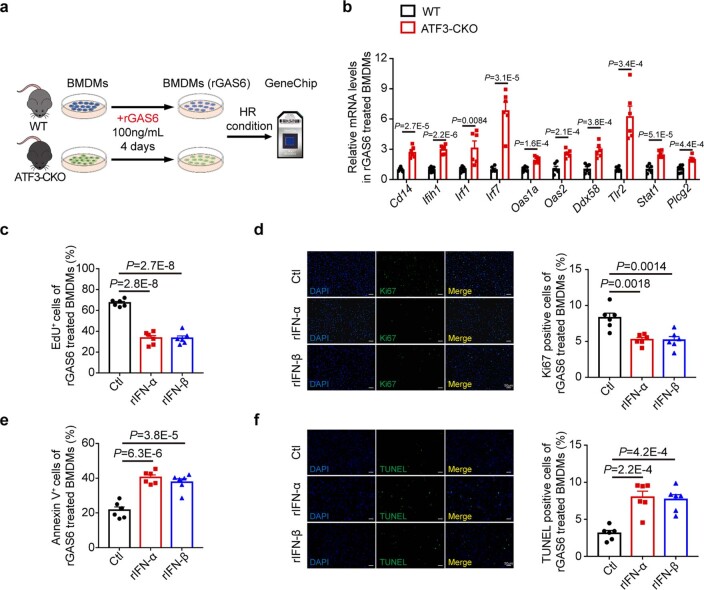
Effect of Type I IFN on MerTK^+^ macrophage *in vitro*. **a**. Schematic representation of the in vitro experimental design. BMDMs obtained from WT and ATF3-CKO mice treated with rGAS6 for 4 days before hypoxia/reoxygenation, followed by GeneChip transcriptome analysis. **b**. Relative mRNA expression of randomly selected type I IFN-related genes in BMDMs obtained from ATF3-CKO and WT mice after HR (n = 6 mice per group). **c**–**f**. Quantification or representative images of EdU^+^ (**c**), Ki67^+^ (**d**), Annexin V^+^ (**e**), and TUNEL^+^ (**f**) cells in rGAS6-treated BMDMs stimulated with rIFN-α or rIFN-β (n = 6 biologically independent samples per group). Scale bar, 50 μm. Statistical significance was evaluated using two-tailed unpaired *t* tests (**b**), and one-way ANOVA analysis followed by Tukey’s multiple comparisons test (**c**–**f**). All data are presented as mean ± SEM. Source data

**Extended Data Fig. 5:**
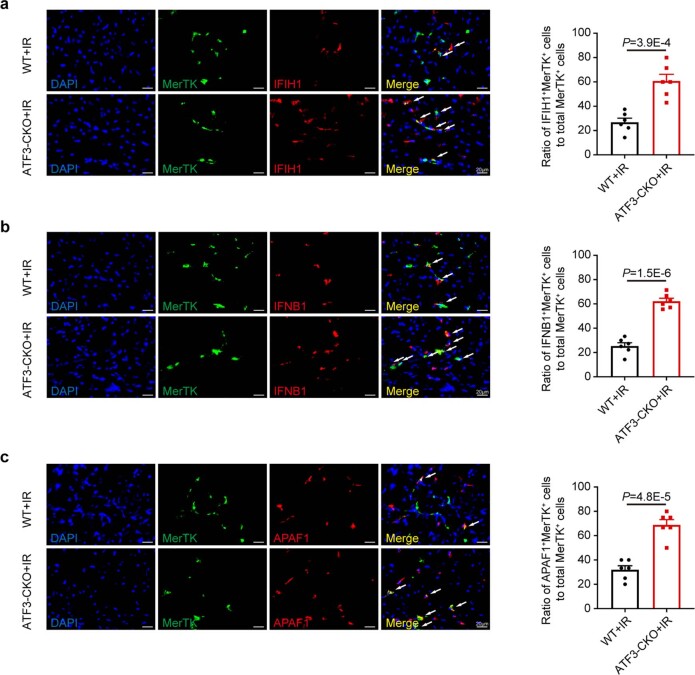
Expression of *Ifih1*, *Ifnb1*, and *Apaf1* in MerTK^+^ cardiac macrophage. **a**–**c**. Representative IF images and quantification of IFIH1^+^MerTK^+^ (**a**), IFNB1^+^MerTK^+^ (**b**) and APAF1^+^MerTK^+^ (**c**) cells in cardiac ischemic regions of WT and ATF3-CKO mice post-IR. Percentage of double positive cells in total MerTK^+^ cells per HPF (n = 6 mice per group). Statistical significance was evaluated using two-tailed unpaired *t* tests (**a**–**c**). All data are presented as mean ± SEM. Source data

We determined the mechanism by which ATF3 regulated the survival and/or proliferation of MerTK^+^ cardiac macrophages. First, we performed bulk RNA-seq to identify transcriptional differences in MerTK^+^ macrophages from WT and ATF3-CKO mice under the HR state (Extended Data Fig. 4a). Notably, gene-set enrichment analysis (GSEA) analysis revealed that type I interferon (IFN) signals, including the response to IFNα, response to IFNβ and positive regulation of type I IFN production, were significantly enriched in ATF3-deficient MerTK^+^ macrophages (Fig. 3a,b and Supplementary Table 2). Validation via RT–qPCR showed higher expression of type I IFN genes in ATF3-deficient MerTK^+^ macrophages than the WT macrophages (Extended Data Fig. 4b). Type I IFN signal plays an important role in controlling proliferation and programmed cell death^17,18^. We postulated that increased type I IFN exerts antiproliferative and proapoptotic effects on MerTK^+^ cardiac macrophages. The rGAS6-induced BMDMs were sorted into MerTK^+^ macrophages then stimulated with recombinant (r)IFNα or rIFNβ. Both rIFNα and rIFNβ increased the apoptosis and suppressed the proliferation of sorted MerTK^+^ macrophages (Fig. 3c,d). Similarly, rIFNα and rIFNβ stimuli reduced proliferation and increased apoptosis in unsorted rGAS6-treated MerTK^+^ macrophages (Extended Data Fig. 4c–f). We next examined whether ATF3 regulates type I IFN gene expression by binding to gene loci in macrophages via chromatin immunoprecipitation sequencing (ChIP–seq). In total, 10.23% of the peaks were mapped to the promoter transcription start site (<3 kb) combined regions (Fig. 3e). When focusing on the genes involved in type I IFN signals, we found that ATF3 bound to a small fraction of differentially expressed genes (DEGs; fold-change >2) between the lentivirus-control (LV-Con)- and LV-ATF3-transfected macrophages. These genes included *Ifih1*, *Ifnb1*, *Ifi47* and *Ifi202b* (Fig. 3f and Supplementary Table 3). We also noticed that ATF3 was bound to *Apaf1*, a well-established proapoptotic gene. An independent ChIP–PCR assay showed that ATF3 could directly bind to *Ifih1* (which encodes MDA5, an important cytoplasmic receptor that senses triggering of IFN production), *Ifnb1 (*which encodes IFNβ*)* and *Apaf1* (which encodes APAF1, a cytoplasmic protein that initiates apoptosis; Fig. 3g). The expression of *Ifih1*, *Ifnb1* and *Apaf1* decreased in LV-ATF3-transfected macrophages but increased in ATF3-deficient macrophages (Fig. 3h,i). The expression of ATF3 target genes was lower in MerTK^+^ macrophages than in MerTK^−^ macrophages sorted from the heart in WT mice after IR; however, their expression was significantly elevated in MerTK^+^ macrophages but not MerTK^−^ macrophages from the heart in ATF3-CKO mice compared with WT mice (Fig. 3j). Beyond RNA levels, we observed that these proteins localized in MerTK^+^ macrophages were higher in ATF3-CKO mice than in WT mice (Extended Data Fig. 5a–c).

**Extended Data Fig. 6:**
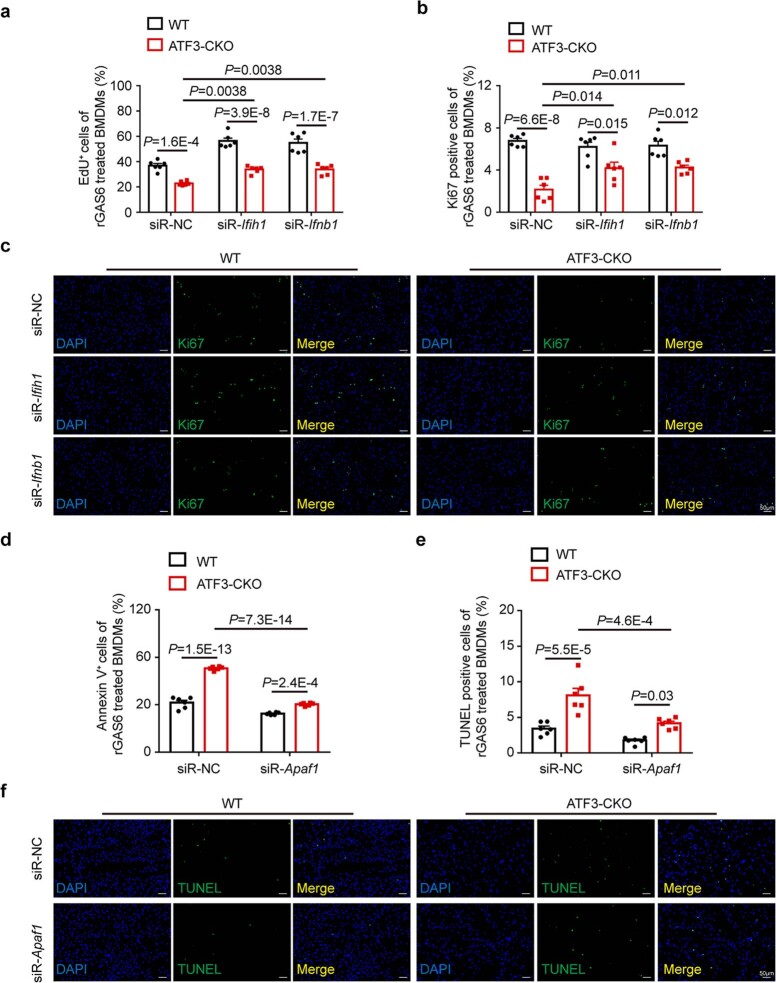
Effect of *Ifih1*, *Ifnb1*, and *Apaf1* on MerTK^+^ macrophage *in vitro*. **a**–**f**. Quantification or representative images of EdU^+^ (**a**), Ki67^+^ (**b**, **c**), Annexin V^+^ (**d**), and TUNEL^+^ (**e**, **f**) cells in rGAS6-treateded BMDMs from ATF3-CKO and WT mice after siRNA transfection (n = 6 biologically independent samples per group). Scale bar, 50 μm. Statistical significance was evaluated using two-tailed two-way ANOVA analysis followed by Tukey’s multiple comparisons test (a, b, d, and e). All data are presented as mean ± SEM. Source data

We tested whether ATF3 regulates macrophage survival and/or proliferation via *Ifih1*, *Ifnb1* and *Apaf1*. In response to HR, BMDMs obtained from ATF3-CKO mice showed increased apoptosis and decreased proliferation compared with those obtained from WT mice, and knockdown of *Ifih1* or *Ifnb1* restored the proliferation and knockdown of *Apaf1* and decreased apoptosis, especially in ATF3-deficient BMDMs (Extended Data Fig. 6a–d). Next, ATK3-CKO mice were treated with an IFNα receptor (AR) neutralizing antibody (NAb) at 0 and every 2 d after IR (Fig. 3k). Immunoglobulin (Ig)G treatment served as the control. The production of IFN-stimulated response element-dependent genes (*Ifit1, Ifit2* and *Ifit3*) was decreased in the heart of IFNAR NAb-treated mice (Fig. 3l). Blockage of IFNAR increased the number of MerTK^+^ macrophages by increasing their proliferation and decreasing their apoptosis (Fig. 3m–p). These data suggested that ATF3 promoted MerTK^+^ cardiac macrophage survival and/or proliferation by inhibiting type I IFN.

### Cardiac macrophage ATF3 deletion hampers cardiac repair

**Fig. 4:**
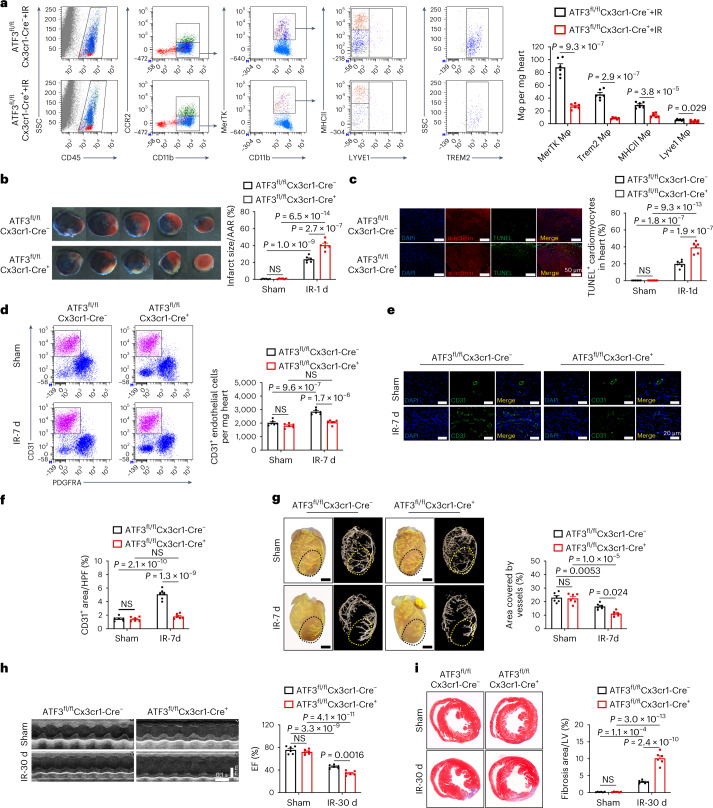
Effect of cardiac resident, macrophage-specific ATF3 on cardiac injury and repair after IR. **a**, Flow cytometry analysis (left) and quantification (right) of MerTK^+^, Trem2^+^, Lyve1^+^ and MHCII^+^ macrophages from the heart of ATF3^fl/fl^Cx3cr1-Cre^−^ and ATF3^fl/fl^Cx3cr1-Cre^+^ mice 6 h after IR (*n* = 6 mice per group). **b**, Representative images of Evans Blue and 2,3,5-triphenyltetrazolium chloride staining in the heart of mice with two genotypes 1 d after IR. Infarct size was calculated as a percentage of the myocardial area at risk (*n* = 6 mice per group). **c**, Representative images (left) and quantification (right) of TUNEL^+^α-actinin^+^ cardiomyocytes in the heart of mice with the two genotypes 1 d after IR (*n* = 6 mice per group). Scale bars, 50 μm. **d**, Flow cytometry analysis (left) and quantification (right) of CD45^−^PDGFRα^−^CD31^+^ ECs in the hearts of mice with the two genotypes (*n* = 6 mice per group). **e**,**f**, Representative IF images (**e**) and quantification (**f**) of CD31^+^ cells in the cardiac border area of mice with the two genotypes (*n* = 6 mice per group). Scale bars, 20 μm. **g**, Representative images (left) and quantification (right) of Microfil vascular casting and micro-CT in the hearts of mice with the two genotypes 7 d after IR (*n* = 6 mice per group). Scale bars, 1 mm. **h**, Representative echocardiographic images (left) and EF (right) of mice with the two genotypes 30 d after IR (*n* = 6 mice per group). **i**, Representative images (left) and quantification (right) of cardiac fibrosis of mice with the two genotypes 30 d after IR (*n* = 6 mice per group). Scale bar, 1 mm. Statistical significance was evaluated using two-tailed, unpaired Student’s *t*-test (**a**) and two-way ANOVA followed by Tukey’s multiple-comparison test (**b**–**d** and **f**–**i**). All data are presented as mean ± s.e.m. Source data

**Extended Data Fig. 7:**
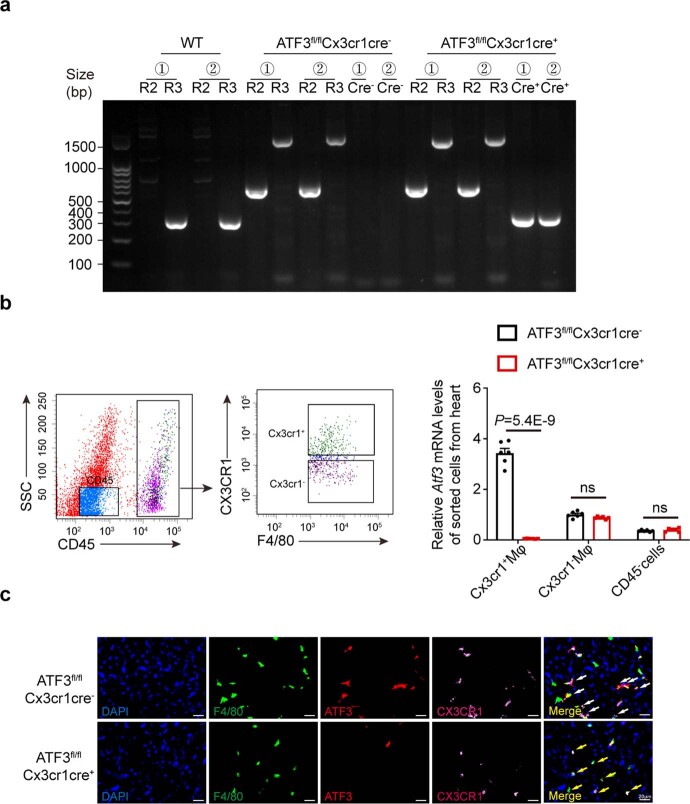
Identification of cardiac resident macrophage-specific ATF3 deletion. **a**. Representative agarose gel images of PCR results using DNA extracted from the tail as a template in ATF3^fl/fl^Cx3cr1-cre^+^ and ATF3^fl/fl^Cx3cr1-cre^-^ mice. **b**. Representative dot plots showing sorting strategy of CD45^−^ cells, Cx3cr1^−^ macrophages, and Cx3cr1^+^ macrophages. Relative mRNA expression of *Atf3* in three types of cells in two genotypes (n = 6 mice per group). **c**. Representative IF images of F4/80 (green), ATF3 (red) and CX3CR1 (pink) in the hearts of the two genotypes. Scale bar, 20 μm. Statistical significance was evaluated using two-tailed unpaired *t* tests (b). All data are presented as mean ± SEM. Source data

**Extended Data Fig. 8:**
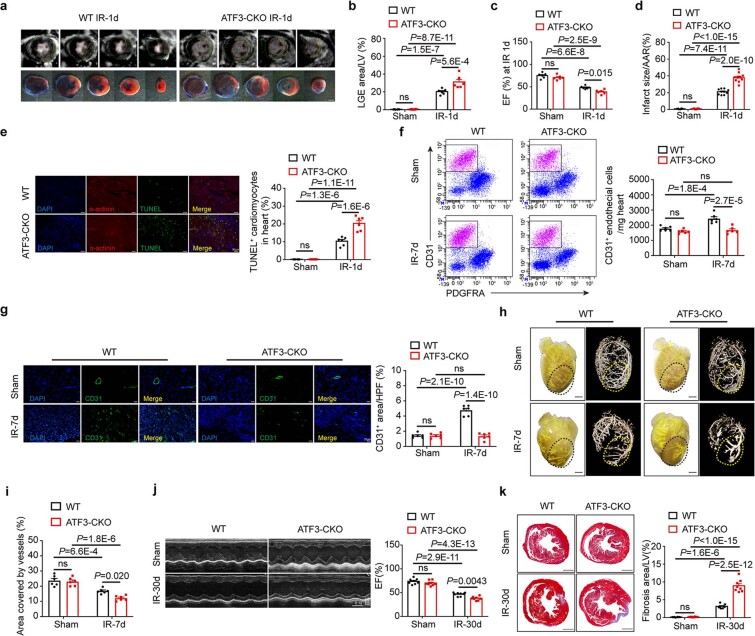
Cardiac repair is attenuated upon the loss of myeloid-specific ATF3. **a**. Representative transverse cardiac magnetic resonance imaging (MRI). The epicardial border (green), endocardial border (red), and infarct region (yellow) are delineated on a T1 map (up). Representative images of Evans blue and 2,3,5^−^triphenyltetrazolium chloride staining (down) in ATF3-CKO and WT hearts 1 day post-IR (bottom). Scale bar, 1 mm. **b**. Late gadolinium enhancement was used to determine the infarct size (n = 6 mice per group). **c**. Ejection fraction (EF) as determined via MRI 1 day post-IR (n = 6 mice per group). **d**. Infarct size was calculated as a percentage of the myocardial area at risk (sham, n = 6 mice per group, IR-1d, n = 10 mice per group). **e**. Representative images and quantification of TUNEL^+^α-actinin^+^ cardiomyocytes in the hearts of ATF3-CKO and WT mice 1 day post-IR (n = 6 mice per group). Scale bar, 50 μm. **f**. Flow cytometry analysis and quantification of CD45^-^PDGFRα^-^CD31^+^ EC populations in the hearts of ATF3-CKO and WT mice (n = 6 mice per group). **g**. Representative images and quantification of CD31^+^ area per HPF in CD31^+^ cells in the cardiac border area of ATF3-CKO and WT mice (n = 6 per group). Scale bar, 20 μm. **h**, **i**. Representative images (**h**) and quantification (**i**) of microfil vascular casting and microCT in ATF3-CKO and WT hearts 7 days after IR (n = 6 mice per group). Scale bar, 1 mm. **j**. Representative echocardiographic images and ejection factor of ATF3-CKO and WT mice 30 days after IR (n = 6 mice per group). **k**. Representative images and quantification of fibrosis of ATF3-CKO and WT hearts 30 days after IR (n = 6 mice per group). Scale bar, 1 mm. Statistical significance was evaluated using two-tailed two-way ANOVA analysis followed by Tukey’s multiple comparisons test (b-g, and i-k). All data are presented as mean ± SEM. Source data

To evaluate the role of ATF3 in cardiac resident macrophage and IR injury/repair, ATF3^fl/fl^ mice were crossed with Cx3cr1-CreTg mice that expressed Cre recombinase under the control of the Cx3cr1 promoter (Extended Data Fig. 7a). ATF3 mRNA expression decreased in sorted cardiac CX3CR1^+^ macrophages, with preserved ATF3 levels in CX3CR1-CD45^+^ cells and CD45^−^ noninflammatory cells compared with ATF3^fl/fl^Cx3cr1-Cre^−^ mice (Extended Data Fig. 7b). Immunostaining shows that ATF3 is absent in CX3CR1^+^F4/80^+^ macrophages in the hearts of ATF3^fl/fl^Cx3cr1-Cre^+^ mice after IR (Extended Data Fig. 7c). ATF3^fl/fl^Cx3cr1-Cre^+^ mice and ATF3^fl/fl^Cx3cr1-Cre^−^ (control group) were subjected to the sham operation or IR. Compared with the control mice, ATF3^fl/fl^Cx3cr1-Cre^+^ mice exhibited a reduction of MerTK^+^ macrophages 6 h after IR, including the Lyve1, MHCII (major histocompatibility complex class II) and Trem2 clusters (Fig. 4a). Infarcted myocardium and apoptotic cardiomyocytes, as determined by Evans Blue and 2,3,5-triphenyltetrazolium chloride staining and TUNEL staining, were markedly increased in ATF3^fl/fl^Cx3cr1-Cre^+^ mice compared with the control mice 1 d after IR (Fig. 4b,c). After the initial cardiomyocyte injury, the post-IR phase was characterized by a reparative stage in which angiogenesis occurred. At 7 d, CD31^+^ ECs were significantly reduced in ATF3^fl/fl^Cx3cr1-Cre^+^ mice compared with those in the control mice (Fig. 4d–f). Microfil vascular casting and micro-computed tomography (micro-CT) imaging showed a reduction in coronary artery area and branching in ATF3^fl/fl^Cx3cr1-Cre^+^ mice compared with those in control mice after IR (Fig. 4g). Ejection fraction (EF) and cardiac fibrosis increased in ATF3^fl/fl^Cx3cr1-Cre^+^ mice (Fig. 4h,i). Under sham-operated conditions, there were no significant differences in heart pathology or function between the two genotypes. We also evaluated heart pathology or function after IR in ATF3-CKO mice. Cine magnetic resonance imaging (MRI) analyses demonstrated that the extent of infarcted myocardium and heart dysfunction was exacerbated in ATF3-CKO mice 1 d after IR (Extended Data Fig. 8a–d). ATF3-CKO mice exhibited more apoptotic cardiomyocytes, less angiogenesis, poor EF and larger fibrosis in the ATF3-CKO mice than in the WT mice after IR (Extended Data Fig. 8e–k). These results indicated that cardiac macrophage-specific ATF3 was required for limiting cardiac injury and promoting repair during IR.

### MerTK^+^ macrophage transfer rescues impaired cardiac repair

**Fig. 5:**
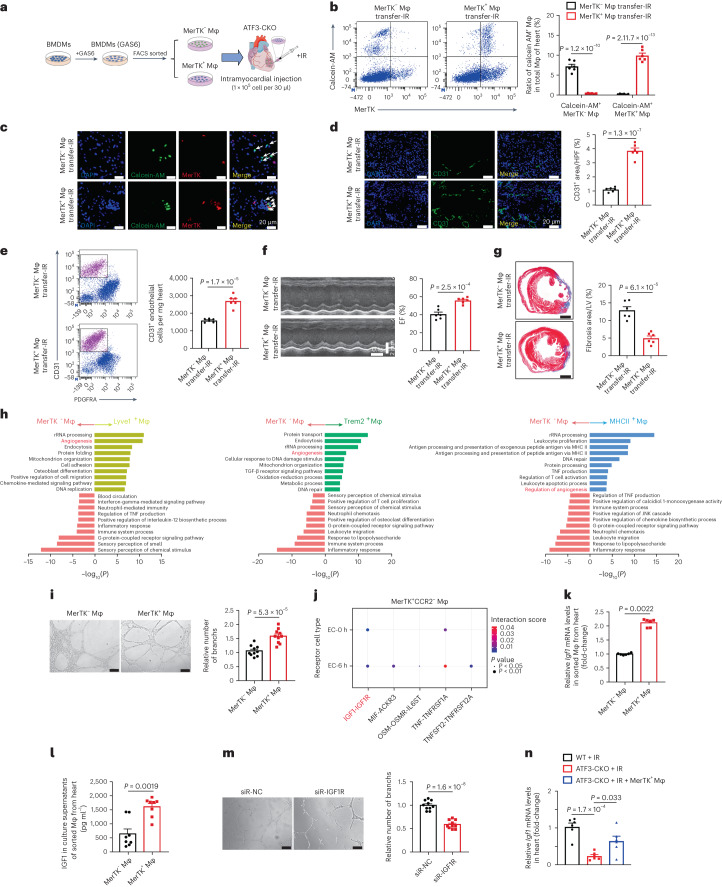
MerTK^+^ macrophage transfer restores cardiac repair in ATF3-CKO mice. **a**, Scheme showing sorted MerTK^−^and MerTK^+^ macrophages transferred into ATF3-CKO mice via intramyocardial injection. **b**,**c**, Flow cytometry analysis (**b**) and immunostaining (**c**) of Calcein AM^+^MerTK^−^ and Calcein AM^+^MerTK^+^ macrophages in hearts of ATF3-CKO mice transferred with MerTK^−^ or MerTK^+^ macrophages (*n* = 6 mice per group). Scale bars, 20 μm. **d***–***g**, Immunostaining (**d**) and flow cytometry analysis (**e**) of CD31^+^ ECs, EF (**f**) and fibrosis (**g**) in the hearts of the two groups (*n* = 6 mice per group). **h**, GO enrichment analysis based on upregulated DEGs in sorted Trem2^+^, MHCII^+^ and Lyve1^+^ macrophages compared with MerTK^−^ macrophages. **i**, Tube formation assay in ECs cocultured with sorted MerTK^+^ and MerTK^−^ macrophages (*n* = 10 biologically independent samples per group). Scale bar, 100 μm. **j**, Predicted interactions between MerTK^+^ cardiac macrophage-derived ligands and receptors expressed on ECs using CellChat receptor–ligand interaction analysis. **k**, Relative mRNA expression of *Igf1* in sorted MerTK^+^ and MerTK^−^ macrophages from hearts after IR (*n* = 6 mice per group). **l**, IGF1 levels in the culture supernatants of sorted MerTK^+^ and MerTK^−^ macrophages from the hearts via ELISA (*n* = 8 biologically independent samples per group). **m**, Tube formation assay in ECs transfected with siRNA-NC or siRNA-*IGF1R* and then cocultured with sorted MerTK^+^ macrophages (*n* = 10 biologically independent samples per group). Scale bars, 100 μm. **n**, Relative mRNA levels of *Igf1* in hearts from WT mice, ATF3-CKO mice and ATF3-CKO mice injected with MerTK^+^ macrophages (*n* = 6 mice per group). Statistical significance was evaluated using two-tailed, unpaired Student’s *t*-tests (**d**–**g**,**i**,**m**), unpaired Mann–Whitney *U*-tests (**k**,**l**), one-way ANOVA followed by Tukey’s multiple-comparison test (**n**), two-way ANOVA followed by Tukey’s multiple-comparison test (**b**) and Fisher’s exact test (**h**,**j**). All data are presented as mean ± s.e.m. Source data

**Extended Data Fig. 9:**
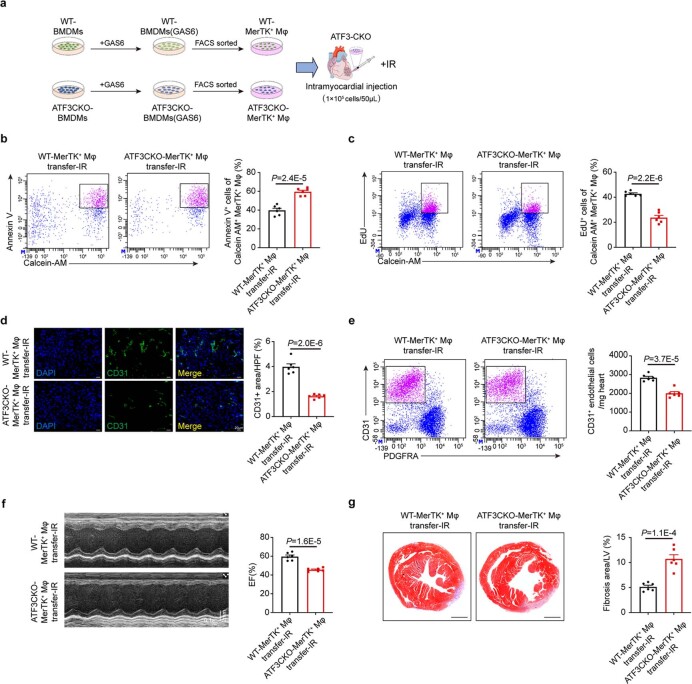
Effect of ATF3 deficient MerTK^+^ macrophage transfer on cardiac repair post IR. **a**. Scheme showing WT-MerTK^+^ and ATF3 deficient-MerTK^+^ macrophages transferred into ATF3-CKO mice. **b**, **c**. Flow cytometry analysis of Annexin V^+^ cells (**b**) and EdU^+^ cells (**c**) gated on Calcein-AM^+^MerTK^+^ macrophages in the heart of mice transferred with two types of MerTK^+^ macrophages (n = 6 mice per group). **d**–**g**. Immunostaining (**d**) and flow cytometry analysis (**e**) of CD31^+^ cells in the cardiac border area, EF (**f**), and cardiac fibrosis (**g**) in the heart of ATF3-CKO mice injected with WT-MerTK^+^ and ATF3 deficient-MerTK^+^ macrophages (n = 6 mice per group). Statistical significance was evaluated using two-tailed unpaired *t*-test (**b**–**g**). All data are presented as mean ± SEM. Source data

We assessed a possible causal relationship between the excessive loss of MerTK^+^ macrophages and poor cardiac repair in ATF3-CKO mice. BMDMs from WT mice were treated with rGAS6 and sorted into MerTK^+^ and MerTK^−^ macrophages, which were then transferred into ATF3-CKO mice via intramyocardial injection (Fig. 5a). Flow cytometry and IF verified macrophage engraftment based on the observed Calcein AM^+^MerTK^+^ or Calcein AM^+^MerTK^−^ macrophage population in the heart 1 d after IR (Fig. 5b,c). We observed fewer Calcein AM^−^MerTK^+^ endogenic macrophages in the heart of MerTK^−^ macrophage-transferred mice. Compared with Ly6C^low^MerTK^+^ cells, Ly6C^hi^MerTK^−^ cells produced more interleukin (IL)-1β and tumor necrosis factor (TNF)-α^19^, which in turn aggravated the death of endogenic macrophages. Of note, injection of MerTK^+^ macrophages improved angiogenesis and heart function and decreased fibrosis compared with MerTK^−^ macrophage injection 7 d after IR in ATF3-CKO mice (Fig. 5d–g). To confirm the role of ATF3 in MerTK^+^ macrophage-mediated protection, BMDMs from WT and ATF3-CKO mice were primed with rGAS6 and then sorted MerTK^+^ macrophages were transferred into ATF3-CKO mice (Extended Data Fig. 9a). More Annexin^+^ cells and fewer EdU^+^ cells were observed in Calcein AM^+^ macrophages in mice with ATF3-deficient MerTK^+^ macrophage transfer (Extended Data Fig. 9b,c). After the transfer of ATF3-deficient MerTK^+^ macrophages, the mice exhibited less angiogenesis, worse heart function and greater fibrosis after IR (Extended Data Fig. 9d–g). These results indicated that ATF3 is required for the survival of MerTK^+^ macrophages, which in turn promotes cardiac repair after IR.

We then analyzed the potential reason for the protective role of MerTK^+^ cardiac macrophages by performing bulk RNA-seq on isolated Trem2^+^, MHCII^+^, Lyve1^+^ and MerTK^−^ macrophages from the heart tissue of WT mice after IR. Pathway analysis was performed by individually comparing the three MerTK^+^ cardiac macrophage clusters with the MerTK^−^ cluster. In addition to endocytosis, we found that the angiogenic pathway was upregulated in the three MerTK^+^ cardiac macrophage clusters (Fig. 5h). We tested the angiogenic ability of sorted MerTK^+^ and MerTK^−^ macrophages by ex vivo coculture with ECs. MerTK^+^ macrophages promoted tube formation in ECs compared with MerTK^−^ macrophages (Fig. 5i). We explored the potential factors of MerTK^+^ cardiac macrophages in the promotion of angiogenesis. Ligand–receptor interaction analysis revealed that insulin-like growth factor 1 (IGF1)–IGF1 receptor (IGF1R) signaling may induce angiogenesis (Fig. 5j). IGF1 expression at both mRNA and protein levels was higher in sorted MerTK^+^ cardiac macrophages than that in MerTK^−^ macrophages (Fig. 5k,l). When blocking IGF1R on ECs via RNA interference, MerTK^+^ cardiac macrophage-induced tube formation was blunted (Fig. 5m). We further observed that cardiac IGF1 expression significantly decreased in ATF3-CKO mice compared with that in WT mice after IR whereas IGF1 expression increased after MerTK^+^ macrophage transfer (Fig. 5n). This result indicates the ability of MerTK^+^ cardiac macrophages to promote angiogenesis by secreting IGF1.

### Protection against IR injury on GAS6 is dependent on ATF3

**Fig. 6:**
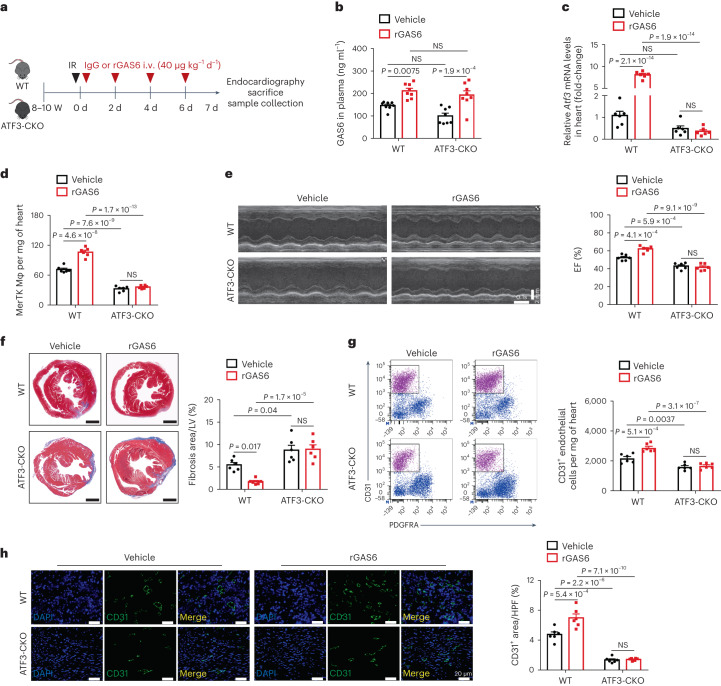
Protective effect of rGAS6 administration against IR injury is dependent on ATF3. **a**, Outline of rGAS6 (40 μg kg^−1^ d^−1^) or vehicle (IgG) treatment in WT or ATF3-CKO mice subjected to IR surgery. **b**–**h**. Plasma GAS6 levels (**b**), cardiac *Atf3* mRNA expression (**c**), MerTK^+^ cardiac macrophages (**d**), EF (**e**), cardiac fibrosis (**f**) and CD31^+^ ECs (**g** and **h**) in WT or ATF3-CKO mice administered rGAS6 (**g**) or IgG (**h**) after IR (**b**, *n* = 8 mice per group; **c**–**h**, *n* = 6 mice per group). Statistical significance was evaluated using two-tailed, two-way ANOVA followed by Tukey’s multiple-comparison test (**b**–**h**). All data are presented as mean ± s.e.m. Source data

The above results led us to hypothesize that enhanced ATF3 expression on GAS6 supplementation would be beneficial for improving cardiac function. We performed a basic, proof-of-principle experiment to evaluate the therapeutic potential of GAS6 during IR. Treatment with commercial rGAS6 or vehicle (IgG) was performed for 7 d (Fig. 6a). The rGAS6-treated mice had higher plasma GAS6 levels than the vehicle group in both WT and ATF3-CKO mice (Fig. 6b). ATF3 expression and MerTK^+^ cardiac macrophage numbers were increased compared with the vehicle group 1 d after IR (Fig. 6c,d). Remarkably, rGAS6-treated mice showed decreased cardiac fibrosis and systolic dysfunction than vehicle-treated mice 7 d after IR (Fig. 6e,f). This ameliorating effect was associated with increased angiogenesis (Fig. 6g,h). Notably, this rGAS6-mediated protection was abolished in ATF3-CKO mice, characterized by the lack of an increase in MerTK^+^ cardiac macrophage proportions, cardiac angiogenesis and cardiac function, between rGAS6 and vehicle treatments (Fig. 6d–h). These results indicated that rGAS6 supplementation could be a protective strategy against cardiac IR injury that relies on the presence of ATF3.

### GAS6–ATF3 is associated with MACEs in patients with ischemia

**Fig. 7:**
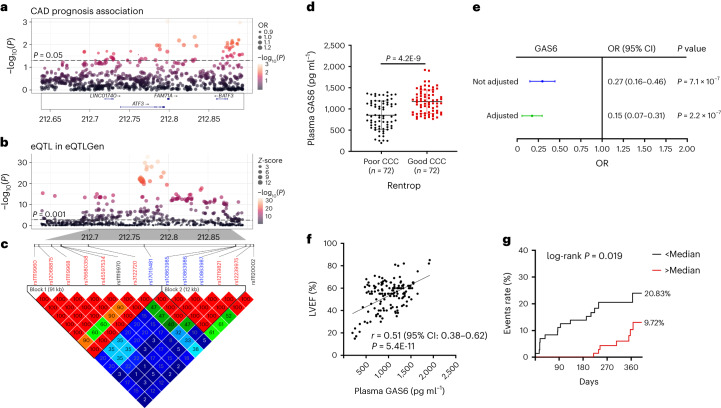
ATF3–GAS6 levels associated with the risk of MACEs in patients with ischemic heart disease. **a**, Manhattan plot indicating GWASs highlighting SNPs in the 100-kb upstream and downstream of ATF3 correlated with the MACEs in patients with CAD. **b**, The eQTLs of ATF3 in the whole blood. **c,** Heatmap of pairwise linkage disequilibrium measurements for the 14 intersection SNPs. **d**, Plasma levels of GAS6 in patients with ischemia with poor or good CCC (*n* = 72 per group). **e**, Unadjusted and adjusted ORs obtained using a logical regression analysis for poor CCC. They were adjusted for drinking and CABG history. **f**, Pearson’s correlation between the plasma GAS6 and LVEF. **g**, Kaplan–Meier plots of major adverse events according to median values of plasma GAS6 levels at admission. Statistical significance was evaluated using two-tailed, unpaired Student’s *t*-tests (**d**), logistic regression analyses (**a**,**e**), Pearson’s correlations analysis (**b**,**f**) and log(rank) tests (**g**). OR, odds ratio. Source data

We further evaluated the impact of GAS6–ATF3 on the progression of ischemic heart disease in humans. First, we interrogated SNPs located in the 100-kb upstream and downstream regions of ATF3 from the UK Biobank database and performed association studies between these SNPs and major adverse cardiac events (MACEs) in 39,707 individuals with coronary artery disease (CAD). The results identified 76 SNPs with *P* < 0.05 (Fig. 7a). The expression quantitative trait locus (eQTL) analysis identified 256 SNPs associated with ATF3 expression in the whole blood (*P* < 0.001) in the eQTLGen database (Fig. 7b). There were 15 SNPs at the intersection of MACE-associated SNPs and ATF3 eQTL variants (Supplementary Table 4). Except for rs3125289, which was not observed in the reference, the linkage disequilibrium of the other 14 SNPs was shown in Fig. 7c. These SNPs were located in two blocks: eight SNPs (including rs11119960, rs12068875, rs11119968, rs45597534, rs3122720, rs12119821 and rs12239975) correlated with increased ATF3 levels were enriched in patients with CAD without MACEs and four SNPs (rs17019481, rs10863985, rs10863986 and rs10863987) correlated with decreased ATF3 levels were enriched in patients with CAD with MACEs (Supplementary Table 4).

We then investigated the association between GAS6 levels and coronary collateral formation and the risk of MACEs in patients with acute coronary syndrome (ACS) and a previous MI. We did not include patients with first-time ACS because the formation of artery-derived collaterals in injured adult hearts takes a long time^20^. The degree of coronary collateral circulation (CCC) was classified as poor and good using the Rentrop score. The baseline demographic and clinical characteristics of matched patients stratified by CCC and healthy controls (HCs) have been reported (Supplementary Table 5). Plasma GAS6 levels were significantly increased in 144 patients with ACS compared with that of 72 HCs (1,013 ± 346 pg ml^−1^ versus 730 ± 121 pg ml^−1^, *P* < 0.001) and ACS patients with good CCC had higher levels of GAS6 than those with poor CCC (Fig. 7d). The area under the curve was 0.76 (95% CI, 0.68–0.84) for plasma GAS6 levels to distinguish good and poor CCC. Logistic regression analysis showed that high GAS6 levels were independently associated with good CCC after adjustment (Fig. 7e). Left ventricular EF (LVEF) was positively correlated with plasma GAS6 levels (Fig. 7f). The patients with high GAS6 levels (≥1,055.8 pg ml^−1^, median values) were less likely to experience MACEs during follow-up (9.72% versus 20.83%; Fig. 7g). Taken together, these integrative human genomic and quantitative protein analyses provided important evidence for the association of GAS6–ATF3 with the risk of MACEs in patients with heart ischemia.

## Discussion

Optimal therapeutic strategies targeting MerTK^+^ cardiac macrophages require a deeper understanding of the regulatory mechanisms governing their fate under ischemic conditions. Our findings demonstrate a transcriptional mechanism that regulates the survival and/or proliferation of MerTK^+^ cardiac macrophages after IR. We showed that MerTK^+^ cardiac macrophages undergo apoptosis in the early response to IR. GAS6-inducible ATF3 is a key regulator of MerTK^+^ cardiac macrophage survival and/or proliferation that represses the expression of type I IFN and proapoptotic *Apaf1*. ATF3 deficiency in cardiac macrophages or myeloid cells results in the remarkable loss of MerTK^+^ cardiac macrophages and their proangiogenic properties, hampering cardiac repair. In the present study, GAS6 treatment improved cardiac repair in an ATF3-dependent manner.

We observed that MerTK^+^ cardiac macrophages displayed a more apoptotic and less proliferative status after IR. An imbalance in apoptosis and proliferation leads to an acute loss of MerTK^+^ cardiac macrophages and hampers cardiac repair. The most striking finding was the identification of ATF3 as a key regulator of the survival and/or proliferation of MerTK^+^ cardiac macrophages in the heart after IR. In our study, we first focused on a panel of TFs (ATF3, c-Fos and Jun) predicted to interact with the signature genes of MerTK^+^ cardiac macrophages based on an in silico analysis. Experiments using siRNAs showed that ATF3 knockdown markedly decreased the proliferation of MerTK^+^ cardiac macrophages and increased their apoptosis. ATF3-CKO mice showed increased apoptosis and reduced proliferation of MerTK^+^ cardiac macrophages, resulting in poor angiogenesis and repair. We also demonstrated that GAS6–MerTK signals induced ATF3 in MerTK^+^ cardiac macrophages. Thus, we provide in vitro and in vivo evidence that the GAS6–ATF3 axis plays an essential role in MerTK^+^ cardiac macrophage survival and/or proliferation during IR.

ATF3 is an inducible protein belonging to the ATF/cAMP-responsive element-binding protein (CREB) family and has been implicated in the pathogenesis of cardiac diseases^21^. Dynamic bioinformatics analysis revealed that ATF3 is a key TF related to the immune response to acute MI^22^. Notably, ATF3 plays an important and complicated role in ischemic heart disease as a protector or facilitator. The complexity of ATF3 is related to three aspects: first, ATF3 acts as either a repressor or an activator of transcription via the formation of homodimers or heterodimers; second, ATF3 affects numerous cell types in the cardiovascular system, which underlies its complex effects. Our previous study revealed a cell-specific protective role of ATF3 in heart failure induced by pressure overload^23^. Fibroblast-specific ATF3 protects against heart failure by repressing MAPK-p38 signaling; conversely, cardiac-specific ATF3 promotes cardiac remodeling. Third, ATF3 plays different roles in different phases of cardiac ischemia. ATF3 deficiency abolished the cardioprotective effect of ischemic preconditioning during IR by repressing IL-6 and intercellular adhesion molecule 1 (ICAM-1)^24^, although ATF3-null mice exhibited an improved survival rate after MI^25^. In the present study, we discovered what we believe is an important role for ATF3 in the prevention of IR injury by regulating the survival and/or proliferation of MerTK^+^ cardiac macrophages. Cardiac resident macrophages exert efferocytosis to remove dying cardiomyocytes. MerTK^+^ macrophages secrete IGF1 to promote angiogenesis, which is a crucial process for cardiomyocyte sustainability during ischemia. Both insufficient angiogenesis and suppressed efferocytosis lead to an accumulation of apoptotic cardiomyocytes in macrophage ATF3-deficient mice. ATF3-deficient myeloid cells aggravated the reduction of MerTK^+^ cardiac macrophages, IR injury and heart dysfunction, which could be rescued by transplanting MerTK^+^ cardiac macrophages.

The prosurvival role of ATF3 has been reported in mast cells, in which a possible mechanism for decreased survival of ATF3-null mice could be a defect in proximal multipotential progenitors; however, this defect was not found in macrophages^26^. Another study showed that ATF3 regulates the proliferation of intestinal stem cells by inhibiting JNK activity^27^. However, our study revealed that c-Jun, a substrate of JNK, did not affect the proliferation of MerTK^+^ cardiac macrophages. These factors did not seem to be involved in the enhanced reduction of MerTK^+^ cardiac macrophage proportions that we observed in ATF3-deficient mice. The present study demonstrates that the mechanism by which ATF3 regulates macrophage survival and/or proliferation is mainly related to the inhibition of type I IFN signaling. Our results supported this mechanism for the following reasons: (1) global gene expression profiling showed that type I IFN signaling was the significantly upregulated pathway in ATF3-deficient MerTK^+^ cardiac macrophages on HR; (2) IFNα and IFNβ stimuli directly promoted apoptosis and inhibited proliferation of MerTK^+^ macrophages; conversely, the blockade of type I IFN completely prevented apoptotic death and restored proliferation; and (3) ChIP experiments revealed that ATF3 is a transcriptional repressor of type I IFN in MerTK^+^ cardiac macrophages. Type I IFN is induced not only by viral recognition but also by other stimuli. Indeed, previous studies have reported that hypoxic conditions increase the expression of IFNα/β in rat kidney cells or human muscle cells^28,29^, which is consistent with our results. The modulating effect of type I IFN on cell survival and/or proliferation has been identified and is not limited to macrophages. Recent studies have demonstrated that type I/III IFN directly reduces epithelial proliferation and that type I IFN-mediated antiproliferation depends on the tumor suppressor protein p53 (ref. ^30^). Type I IFN signaling induces the death of myeloid progenitors by dysregulating iron metabolism and depolarizing mitochondrial membrane potential^31^. Furthermore, IFNβ inhibits the survival of group 2 innate lymphoid cells^32^. In addition to its role in type I IFN, ATF3 inhibits the proapoptotic factor Apaf1, which plays a key role in regulating the formation of the apoptotic core machinery, the apoptosome, to induce programmed cell death. *Apaf1*-deficient cells are resistant to various apoptotic stimuli^33^. Multiple genes that are governed by ATF3 may cooperatively contribute to the survival and/or proliferation process. Taken together, our results demonstrate that ATF3 is crucial for maintaining the survival and/or proliferation of MerTK^+^ cardiac macrophages in response to IR.

To the best of our knowledge, this is one of the first reports to demonstrate that the GAS6–ATF3 axis enhances the survival and/or proliferation of MerTK^+^ cardiac macrophages, which promotes cardiac repair via angiogenesis. GAS6 administration during the perioperative period enhances MerTK^+^ cardiac macrophages and angiogenesis, limiting progression to heart failure. Accumulating evidence demonstrates that GAS6 participates in wound healing and exerts protective effects in various IR models. GAS6-deficient mice die within 12 h of hepatic IR because of massive hepatocellular injury. Conversely, GAS6 supplementation rescues null mice from liver IR injury^34^. Treatment with GAS6 leads to a reduction in inflammation and protection against renal injury induced by renal IR^35^. Furthermore, GAS6-engineered, mesenchymal stem cell-treated animals show remarkable cardiac functional recovery after MI compared with control mesenchymal stem cell treatment^36^. GAS6 binds to all three TAM receptors (Tyro3, AXL and MerTK) and has the highest affinity for AXL^37,38^. Most of the protective effects of GAS6 described in different cell types are attributed to the binding of GAS6 to AXL. It is interesting that we found that the cardioprotective role of GAS6 in cardiac IR may depend on MerTK-ATF3. This is supported by the fact that the protective effects elicited by GAS6 against cardiac IR injury are blunted in ATF3-deficient mice. GAS6-induced ATF3 activation in macrophages is dependent on MerTK and downstream AKT signaling. A previous study reported that the binding of GAS6 to MerTK, but not to AXL or Tyro3, resulted in the activation of AKT^34^. Importantly, a recent study showed that AXL, in contrast to MerTK, antagonized cardiac repair after MI. Administration of a selective small-molecule AXL inhibitor improves cardiac healing^39^. In addition to regulating survival and/or growth, GAS6–MerTK signaling crucially assists in maintaining a balance between proinflammatory responses to pathogens and the prevention of autoimmune reactions that could lead to tissue damage^40^. Thus, these studies indicated that GAS6–MerTK signals may elicit combined survival and anti-inflammatory effect to protect against cardiac IR injury.

The present findings have high clinical significance. The integration of summary data from the genome-wide association study and eQTL analyses revealed 12 SNPs that exert regulatory effects on ATF3 expression and are associated with the risk of MACEs in patients with CAD. GAS6 has not been previously proposed as a serological marker of CCC in patients with ischemia. We found that plasma GAS6 levels were significantly correlated with improved CCC, cardiac function and prognosis. This finding is not surprising from a pathophysiological perspective because rGAS6 promotes angiogenesis and cardiac repair by activating ATF3. These clinical data further support the use of GAS6 supplementation as a promising therapeutic tool for IR injury. However, well-designed clinical studies are necessary to test its therapeutic efficacy, safety and potential benefits, as well as its side effects before clinical application.

## Conclusion

In summary, our study addresses previously unanswered questions regarding the cell-intrinsic mechanisms of MerTK^+^ cardiac macrophage fate in response to IR. We revealed a central role of ATF3 in maintaining the survival and/or proliferation of MerTK^+^ cardiac macrophages and identified the GAS6–ATF3 axis as an important factor controlling cardiac repair after IR. This biological insight will aid in the development or improvement of therapies targeting cardiac macrophages for the treatment of ischemic injury and heart failure.

## Methods

### Experimental animals

Conditional deletion of ATF3 in myeloid lineages (ATF3-CKO) or resident cardiac macrophages was achieved by crossing ATF3^flox/flox^ mice with Lyz2-Cre transgenic mice or Cx3cr1-Cre transgenic mice and breeding them to homozygosity. Cre^−^ littermate mice served as the WT control. All experimental animals were maintained on a C57BL/6 genetic background. ATF3^flox/flox^ mice were generated by Cyagen Biosciences, Inc. Genotyping primers for ATF3^flox/flox^ were as follows: forward (F)1, 5′-TTAGTTTGGAAGTGGATGGTGCATG-3′; reverse (R)1, 5′-CGCCCTTGCTCACCATCTATAAAAT-3′; and R2, 5′-CTTGGAACAACTTTACCCATCCCC-3′. Lyz2-Cre (catalog no. 004781) and Cx3cr1-Cre mice (catalog no. 025524) were purchased from Jackson Laboratory. Male C57BL/6 mice were purchased from Viewsolid Biotech (catalog no. VSM10001). Male mice aged 10–12 weeks were used in the experiments. All animals were housed under pathogen-free conditions and allowed free access to food and water. The room was maintained under a controlled temperature (20–25 °C), humidity (30–70%) and light exposure cycle (12-h light and dark cycles) conditions. All experiments involving animals were conducted based on the Guidelines on the Use and Care of Laboratory Animals and approved by the Animal Subjects Committee of Beijing Anzhen Hospital, Capital Medical University.

### Myocardial IR in vivo and in vitro

#### Coronary ligation and reperfusion in mice

The IR procedure was performed as previously described^2^. Briefly, 10- to 12-week-old male mice were anesthetized by 2–3% isoflurane inhalation. The heart was accessed through the third intercostal space on the left side. A slipknot was tied around the descending branch of the left coronary artery (LAD) 3 mm from its origin with a 6/0 silk suture. Ischemia was confirmed by changes in the electrocardiogram (ST-segment elevation) and myocardial color. After 30 min of ischemia, the slipknot was released to achieve reperfusion. Sham-operated mice were subjected to the same procedure without LAD ligation. For GAS6 treatment, murine rGAS6 (R&D, catalog no. 986-GS-025; 40 μg kg^−1^ d^−1^) or IgG was administered to WT or ATF3-CKO mice 30 min before IR and 2, 4 and 6 d after IR. For MerTK^+^ macrophage transfer, we purified MerTK^+^ macrophages from rGAS6-stimulated macrophages of WT or ATF3-CKO mice using FACS. FACS-sorted MerTK^−^ macrophages or MerTK^+^ macrophages (1 × 10^5^) were suspended in 30 µl of phosphate-buffered saline (PBS) and injected intramyocardially at five sites (6 µl per site) immediately after LAD ligation^41,42^. For IFNAR NAb treatment, MAR1-5A3 IFNAR NAb (BioXCell, catalog no. BE0241; 20 mg kg^–1^ d^–1^) or IgG was administered to ATF3-CKO mice 30 min before IR and 2, 4 and 6 d after IR.

#### HR in macrophages

For the HR experiments, BMDMs cultured in serum-free Roswell Park Memorial Institute (RPMI)-1640 medium were transferred to a hypoxic incubator with a humidified atmosphere equilibrated with 95% N_2_ and 5% CO_2_ at 37 °C. After 6 h, BMDMs were reoxygenated by replacing the medium with the complete medium under normoxic conditions (humidified atmosphere of 21% O_2_ and 5% CO_2_) at 37 °C for 18 h. Normoxic control cells were incubated at 37 °C under 21% O_2_ and 5% CO_2_ throughout.

### Cardiac imaging

#### MRI

MI and mouse function were evaluated with cardiac MRI using a small animal MRI system (BioSpec70/20). The mice were anesthetized via inhalation of 2–3% isoflurane mixed with medical-grade O_2_ using a nose cone and were placed in the supine position on a mouse holder. Electrocardiography was continuously performed during the entire MRI procedure. The core temperature was maintained at 33 ± 2 °C to ensure an adequate and constant heart rate. The images were acquired using cine and late gadolinium enhancement imaging pulse sequences. All cardiac MR images were analyzed using dedicated software (QMass MR v.7.6, Medis; ImageJ v.1.4, National Institutes of Health). Infarct size was determined by summing the percentage of myocardial mass enhanced by gadolinium along the serial short-axis sections and quantified at end-diastole for all four sections. The average values were then calculated. Cardiac LV borders were manually traced in each cine image to calculate the LVEF^2^.

#### Echocardiography

Transthoracic echocardiographic analysis was performed on conscious mice after IR using a VisualSonics Vevo 2100 Ultrasound system (VisualSonics) and a 30-MHz linear array transducer. Two-dimensional guided, M-mode measurements of anterior and posterior wall thicknesses at end-diastole and end-systole were recorded. The LV internal diameter was measured as the largest anteroposterior diameter in diastole or systole. LVEF data were calculated as previously described^43^.

### Measurement of the AAR and infarct size

After 24 h of reperfusion, the LAD was reoccluded at the previous site and 2% Evans Blue (Sigma-Aldrich, catalog no. E2129) was injected quickly into the ascending aorta and coronary arteries to demarcate the ischemic area at risk (AAR). The heart was quickly excised and cut into five 1-mm slices. To visualize the infarct size, the slices were then incubated with 1% triphenyltetrazolium chloride solution at 37 °C for 15 min before being imaged under a microscope. The percentages of the area of infarction and AAR of each section were multiplied by the weight of the section and then totaled across all sections. Finally, the infarct size/AAR was expressed as a percentage^44^.

### Induction of MerTK^+^ macrophages in vitro

BMDMs were isolated from the femurs and tibiae of adult mice and differentiated into macrophages in RPMI-1640 medium supplemented with 20 ng ml^−1^ of recombinant murine macrophage colony-stimulating factor (Peprotech, catalog no. 315-02) for 5 d. BMDMs were further treated with rGAS6 (100 ng ml^−1^) for 4 d to induce MerTK^+^ macrophages^45^. GAS6-induced MerTK^+^ macrophages were further exposed to HR conditions, or rIFNα (SinoBiological, catalog no. 50672-M08H, 100 ng ml^−1^) or rIFNβ (SinoBiological, catalog no. 50708-M02H, 100 ng ml^−1^) stimulation.

### TUNEL assay

TUNEL assay was performed on paraffin-embedded heart sections or cultured macrophages to detect apoptosis using the In Situ Cell Death Detection Kit (Roche, catalog no. 11684817910) according to the manufacturer’s protocol. Briefly, samples were permeabilized with 0.1% Triton X-100 and subsequently incubated with the TUNEL reaction solution mixture in a humidified 37 °C chamber for 60 min. The cell nuclei were labeled with DAPI (Abcam, catalog no. ab104139). Images were captured using a fluorescence microscope. TUNEL-positive cells were counted in five randomly selected fields of the slide and expressed as a percentage of total cells.

### Angiogenesis assessment

#### Coronary vascular perfusion and micro-CT

Mice were anesthetized with isoflurane and cannulated retrogradely via the descending thoracic aorta and the vasculature was cleared by perfusion at 100 mmHg pressure for 5 min with saline containing heparin sodium salt (25 U ml^−1^, Sigma-Aldrich, catalog no. H3149). Microfil compounds, specifically MV-122 Compound, MV-Diluent and MV-Curing Agent (Flow Tech, catalog no. MV-122), were mixed at a 10:8:1 ratio and injected directly until the casting compound was perfused throughout the vasculature. After Microfil polymerization and overnight fixation with 4% paraformaldehyde, the hearts were imaged using micro-CT on a Siemens Inveon positron emission tomography/CT scanner and three-dimensional reconstruction of perfused vasculature^46^. Regions of interest (ROIs) were drawn to delineate the ischemic areas in IR mice or equivalent areas in sham-operated mice. Mean ROI values were extracted to analyze vessel coverage in the ischemic area using Inveon Research Workplace software. Branch nodes were quantified as a surrogate measure of vascular complexity.

#### Tube formation assay

A tube formation assay was performed as previously described^47^. Briefly, MerTK^+^ macrophages or MerTK^−^ macrophages sorted from the heart were seeded on individual inserts of a Transwell-24 plate and cultured overnight in RPMI-1640 medium supplemented with 10% fetal bovine serum (FBS; Gibco, catalog no. 10091148) and 1% penicillin–streptomycin (Gibco, catalog no. 15140122). Growth factor-reduced Matrigel (BD Biosciences, catalog no. 354230) was plated at the bottom of a Transwell-24 plate (200 μl per well) after being thawed at 4 °C overnight and polymerized for 1 h at 37 °C. Human umbilical vein endothelial cells (HUVECs) were purchased from American Type Culture Collection (catalog no. CRL-1730) and cultured in EC medium (ScienCell, catalog no. 1001) supplemented with 5% FBS, 1% EC growth supplement and 1% penicillin–streptomycin solution. HUVECs were then seeded in the Matrigel-coated wells (1 × 10^5^ cells per well). After coculturing for 8 h, the total capillary tube branches per field were observed and quantified by counting random fields per well using microscopy.

### Proliferation assessment of MerTK^+^ macrophages

To assess the in vitro proliferation capacity of GAS6-induced MerTK^+^ macrophages, the macrophages were first transfected with the indicated siRNAs, followed by cell culture under HR conditions or stimulation with rIFNα or rIFNβ. After 24 h, EdU (10 µM) was added to the culture medium to label proliferating cells, and the cells were incubated for 2 h. To determine cell proliferation in vivo, ATF3-CKO or WT mice were injected intraperitoneally with 0.1 mg of EdU (100 μl in PBS) 2 h before macrophage collection. The incorporated EdU was visualized by incubating the MerTK^+^ macrophages or digested cardiac cells with Click-iT Plus EdU AF647 Imaging Kit reagents (Thermo Fisher Scientific, catalog no. C10634) according to the manufacturer’s instructions. EdU-positive macrophages were analyzed using flow cytometry to determine the cell proliferation rate^48^.

### Transcriptome analysis

#### ScRNA-seq

LV tissues of three hearts were isolated 6 h after an IR or sham operation, minced with fine scissors and digested using the Neonatal Heart Dissociation Kit (Miltenyi Biotec, catalog no. 130-098-373) in a 37 °C water bath and the gentleMACS Dissociator according to the manufacturer’s protocol. Digested cells were filtered through a 100-μm cell strainer and centrifuged at 300*g* for 10 min to obtain a single-cell suspension. Then, cell debris was removed using Debris Removal Solution (Miltenyi Biotec, catalog no. 130-109-398). The suspension was resuspended in Dulbecco’s PBS containing 0.04% bovine serum albumin (BSA) and loaded into a 10× Chromium Controller (10× Genomics)^49^. RNA from the barcoded cells was subsequently reverse transcribed and sequencing libraries were constructed with reagents from a 10× Chromium Single Cell 30 v.2 reagent kit (10× Genomics) according to the manufacturer’s protocol. The resulting libraries were sequenced on an Illumina NovaSeq6000 platform.

Raw base-call files were converted into FASTQ files using bcl2fastq (2.18.0.12). The 10× CellRanger pipeline was used to align the raw data to the mouse reference transcriptome (mm10) and generate gene-cell count matrices. Initial quality control and clustering were performed using Seurat v.2.2.1. To remove dead or falsely identified cells as well as doublets, genes expressed in <5 cells, cells with <200 or >2,500 detected genes and cells with >30% mitochondrial genes were filtered. Data were normalized using a global scaling method, converted to a scale factor (10,000 by default), and log(transformed). Highly variable genes (HVGs) across individual datasets were identified using the Find Variable Features method by selecting 2,000 genes with the highest feature variance. The ScaleData function was used to scale and center the expression values in the dataset and the number of unique molecular identifiers was regressed against each gene.

After data normalization and scaling to remove unwanted sources of variation, principal component analysis was conducted using genes with a highly variable expression. The cell types were annotated based on marker genes and matched to canonical markers. Nonlinear dimensional reduction (UMAP) and graph-based clustering of single cells merged from both groups identified seven transcriptionally distinct clusters of monocytes/macrophages. We provided the list of maker genes of seven clusters of macrophages/monocytes (Supplementary Table 6). DEGs between clusters were statistically determined using a Wilcoxon’s rank-sum test with a *P* value threshold of 0.05 and a log_2_(fold-change) (log_2_(FC)) >0.25. Functional analysis of DEGs was performed using enrichKEGG in clusterProfiler, with *P* < 0.05.

Clustering was performed using the FindClusters function. Marker genes of each cluster were identified using the FindAllMarkers function. The cell types were annotated based on marker genes and matched to canonical markers. The closeness of the obtained clusters was inferred by hierarchical clustering (hclust function in R), based on the average expression of the HVGs in each cluster^50^. CellChat (1.5.0) was used to study the interactions between cell clusters and to determine the mechanism of action of the communication molecules based on the scRNA-seq data. Using secret signaling in the CellChat database, we identified the ligands and receptors expressed differently in each cell cluster of the two samples and calculated the probability of each interaction according to the mass interaction law. The ligand pairs of interest were described using Dotplot^51^. For TF prediction, marker genes (*P* < 0.05, log_2_(FC) > 0.25) of MerTK^+^CCR2^−^ macrophages were used as input for RCisTarget analysis^52^. The analysis was performed with default parameters following the RCisTarget Vignette.

#### Microarray

Total RNA was extracted from four macrophage clusters (Lyve1^+^ Mφ, Trem2^+^ Mφ, MHCII^+^ Mφ and MerTK^−^ Mφ) sorted from the IR hearts of WT mice, as well as from GAS6-induced MerTK^+^ macrophages of WT or ATF3-CKO mice, and cultured under HR conditions using TRIzol reagent (Invitrogen, catalog no. 15596018) as previously described^23^. RNA was purified using an RNeasy mini kit (QIAGEN, catalog no. 74004) and used to generate amplified and biotinylated complementary DNA using the Ambion WT Expression Kit (Invitrogen, catalog no. 4411973). Fragmented cDNA was hybridized on Clariom S Assay (mouse, Affymetrix) for 16 h in a rotating incubator at 45 °C. Gene chips were washed and stained using the Affymetrix Fluidics Station 450 and scanned using a GeneChip Scanner 3000 7G (Affymetrix). The row data were normalized using the Robust Multichip Analysis (RMA) algorithm with Affymetrix default analysis settings and by employing global scaling as the normalization method. The log_2_(RMA) intensity signals were imported into R (installed with Bioconductor packages) and further processed using the limma package (linear model for microarray analysis, 3.52.4).

The limma package in R was used to calculate fold-changes and *P* values for differential expression using the empirical Bayes statistics for differential expression (eBayes) framework. DEGs were defined as genes with a fold-change ≥2. Gene ontology (GO) analysis was performed using enrichGO included in the R package clusterProfiler by providing the GO gene set retrieved from the Molecular Signatures Database. A GO term was considered significantly enriched if its enrichment had a false discovery rate (FDR) ≤0.05. To identify signaling pathways that are differentially activated between WT and ATF3-CKO MerTK^+^ macrophages, we selected an ordered list of genes according to the fold-change in expression values. GSEA was performed using the GO database (Biological Processes) to elucidate specific biological functions of the resulting gene sets. The resulting GSEA data were analyzed and visualized using the R package clusterProfiler in an enrichment plot. An adjusted *P* value of 0.05 was used as the significance threshold.

### Flow cytometry

LV tissues were isolated, minced with fine scissors and subjected to an enzymatic digestion solution containing collagenase II (200 U ml^−1^, Thermo Fisher Scientific, catalog no. 17101015) and dispase II (1 U ml^−1^, Roche, catalog no. 04942078001) at 37 °C for 30 min. Cells were collected and filtered (40 μm) to generate a single-cell suspension. The cells were then incubated with fluorescently labeled anti-mouse antibodies, namely, PE-CF594-CD45 (BD, catalog no. 562420), BV510-CD11b (BD, catalog no. 562950), phycoerythrin (PE)-Cy7-F4/80 (eBioscience, catalog no. 25-4801-82), PE-F4/80 (BD, catalog no. 565410), PE-Cy7-MerTK (BioLegend, catalog no. 151522), BV650-CCR2 (BioLegend, catalog no. 150613), PE-LYVE1 (eBioscience, catalog no. 12-0443-82), AF700-MHC-II (eBioscience, catalog no. 56-5321-82), BV510-CX3CR1 (BioLegend, catalog no. 149025), PE-CD31 (BD, catalog no. 561073), allophycocyanin (APC)-PDGFR-α (BD, catalog no. 562777), PE-Ly6G (BD, catalog no. 553128), FITC-CD3 (BioLegend, catalog no. 100204), and APC-Cy7-CD19 (BD, catalog no. 561737) or isotype control antibody (BV510 IgG isotype control, BD, catalog no. 562951, PE IgG isotype control, BD, catalog no. 553930 and PE-Cy7 IgG isotype control, BD, catalog no. 552784) at 4 °C for 40 min.

The FITC Annexin V apoptosis detection kit (BD Biosciences, catalog no. 556547) was used following the manufacturer’s protocol. Cardiac cell or BMDM suspensions were incubated with anti-CD45, anti-CD11b, anti-MerTK and anti-CCR2 (all diluted 1:200), resuspended in Annexin V binding buffer (10 mM Hepes, 2.5 mM CaCl_2_ and 140 mM NaCl). Of this cell suspension, 100 μl (1 × 10^5^ cells) was stained with FITC-conjugated Annexin V for 25 min at room temperature in the dark. The cells were washed with staining buffer (PBS containing 0.04% BSA) and analyzed on a BD FACS Aria II flow cytometer (Becton Dickinson) according to the manufacturer’s protocol^53^.

For cell sorting, cardiac cell suspensions or rGAS6-stimulated BMDM suspensions were prepared and incubated with fluorescently labeled anti-mouse antibodies. Heart cells were sorted by gating for CD45^+^CD11b^+^ cells via flow cytometry (Beckman Coulter MoFlo XDP) into populations of MerTK^−^, MerTK^+^, Lyve1^+^, MHCII^+^ and Trem2^+^ macrophages^54^. In addition, to verify the conditional deletion of ATF3, macrophages were gated as CD45^+^CD11b^+^F4/80^+^, fibroblasts were gated as CD45^−^CD11b^−^CD31^−^PDGFR-α^+^, cardiomyocytes were gated as CD45^−^CD11b^−^CD31^−^PDGFR-α^−^, ECs were gated as CD45^−^CD11b^−^CD31^+^PDGFR-α^−^, CX3CR1^−^ macrophages were gated as CD45^+^CD11b^+^F4/80^+^CX3CR1^−^ and CX3CR1^+^ macrophages were gated as CD45^+^CD11b^+^F4/80^+^CX3CR1^+^. GAS6-stimulated BMDMs were sorted into MerTK^−^ macrophages or MerTK^+^ macrophages for intramyocardial injection.

### Immunofluorescence

Formalin-fixed, paraffin-embedded heart sections were deparaffinized and rehydrated for IF. Antigen retrieval was performed in citrate buffer, pH 6.0 or EDTA buffer, pH 9.0 using an 800-W microwave at 100% power for 15 min. The cells were fixed with prechilled methanol for 10 min, washed twice with PBS at 5-min intervals and then permeabilized with 0.1% Triton X-100 for 10 min at room temperature. The sections and cells were blocked with 5% BSA/PBS for 60 min and stained overnight at 4 °C with primary antibodies, namely, anti-MerTK (Abcam, catalog no. ab300136; 1:100), anti-F4/80 (Abcam, catalog no. ab6640; 1:100), anti-Ki67 (Abcam, catalog no. ab15580; 1:100), anti-ATF3 (Abcam, catalog no. ab207434; 1:100), anti-α-actinin (Sigma-Aldrich, catalog no. A7732; 1:100), anti-CD31 (Abcam, catalog no. ab222783; 1:100), anti-Col1A2 (Abcam, catalog no. ab208638; 1:100), anti-TREM2 (Invitrogen, catalog no. PA5-119690; 1:100), anti-LYVE1 (Invitrogen, catalog no. MA5-32512; 1:100), anti-CD74 (Abcam, catalog no. ab289885; 1:100), anti-CX3CR1 (Abcam, catalog no. ab308613; 1:100), anti-IFIH1 (Abcam, catalog no. ab79055; 1:100), anti-IFNB1 (Invitrogen, catalog no. PA5-102429; 1:100) and anti-APAF1 (Invitrogen, catalog no. MA5-32082; 1:100). Next, secondary antibodies conjugated to Alexa Fluor-488 (Invitrogen, catalog nos. A-11001 and A-11008; 1:500), Alexa Fluor-555 (Invitrogen, catalog nos. A-21422 and A-31572; 1:500) or Alexa Fluor-633 (Invitrogen, catalog no. A-21070 1:500) was incubated with the stained sections and cells for 1 h at room temperature in 1% BSA/PBS. The nuclei were stained with DAPI. All immunofluorescence images were captured using a Leica ST5 laser scanning confocal microscope^55^.

### Masson’s Trichrome Stain

The paraffin sections were deparaffinized and rehydrated. After being washed in distilled water, the sections were stained using Masson Trichrome Stain kit (Leagene, catalog no. DC0032). Images of sections were acquired using a light microscope (Nikon Eclipse TE2000-S). The mean percentage of the fibrotic area over the total area was measured and calculated using the ImageJ software.

### RT–qPCR

Total RNA was extracted from tissues and cultured cells using TRIzol reagent (Invitrogen, catalog no. 15596018), followed by chloroform extraction. The cDNA was synthesized from mRNA (2 μg) using a reverse transcription kit (Promega, catalog no. A5000). RT–qPCR was performed on the iCycler iQ system (BioRad) using SYBR Green PCR Master Mix (TaKaRa, catalog no. RR420A) according to the manufacturer’s instructions. The relative expression level of each mRNA was calculated using the 2^–ΔΔ*C*T^ cycle threshold method and was normalized to glyceraldehyde 3-phosphate dehydrogenase (GADPH) mRNA expression. In the RT–qPCR assay, all samples were run in triplicate in every independent experiment to minimize the variation. The RT–qPCR primers are listed in Supplementary Table 7.

### Western blot analysis

Cells were washed twice with PBS and lysed for 30 min on ice in a lysis buffer (T-PER; Thermo Fisher Scientific, catalog no. 78510) containing a protease inhibitor cocktail and phosphatase inhibitors (Thermo Fisher Scientific, catalog no. A32957). The samples were subjected to 10% sodium dodecylsulfate–polyacrylamide gel electrophoresis and transferred to nitrocellulose membranes. The membranes were subsequently blocked with 5% (w:v) skimmed milk and then incubated at 4 °C overnight with the following primary antibodies: anti-phospho-Akt (Cell Signaling Technology, catalog no. 13038; 1:1,000), anti-Akt (Cell Signaling Technology, catalog no. 4691; 1:1,000), anti-phospho-MERTK (Abcam, catalog no. ab192649; 1:1,000), anti-MERTK (Abcam, catalog no. ab300136; 1:1,000), anti-ATF3 (Abcam, catalog no. ab207434; 1:1,000) and anti-GAPDH (catalog nos. ZSGB-BIO and TA08; 1:1,000). After three washes with Tris-buffered saline–Tween 20, the membranes were incubated with infrared dye 800-conjugated secondary antibodies (Rockland Immunochemicals, catalog no. 611-145-002; 1:5,000) for 1 h at room temperature. The blot images were subsequently captured and quantified using an Odyssey infrared imaging system (LI-COR Biosciences)^56^.

### ChIP assays

ChIP assays were performed as previously described^23^. Briefly, BMDMs were transfected with LV-Con or LV-ATF3 for 72 h. BMDMs (4 × 10^6^) were crosslinked with 1% formaldehyde for 10 min at room temperature, neutralized with glycine and lysed, and then the cell nuclei were washed. Chromatin was sonicated to an average size of 0.5–2.0 kb and immunoprecipitated using rabbit anti-ATF3 antibody (Abcam, catalog no. ab207434) or IgG (Abcam, catalog no. ab172730). A small portion of the crosslinked sheared chromatin was saved as an input control. Then, protein A/G Dynal magnetic beads were added to the ChIP reaction mixture and incubated for 4 h at 4 °C. The immunoprecipitates were washed, reverse crosslinked and incubated with proteinase K and RNase A. ChIP DNA was purified via phenol–chloroform and sodium chloride precipitation. The extracted DNA was used for RT–qPCR and sequencing.

For ChIP–seq, the concentration and quality of the DNA fragments were assessed on Qubit Fluorometer and Agilent Bioanalyzer 2100. DNA was end-repaired, followed by the addition of an A base to the 3′-ends, adapter ligation and finally PCR amplification. Ligation products were size selected (175–225 bp) and assessed using Agilent Bioanalyzer. The libraries were pooled and sequenced using the Illumina HiSeq2000 platform. Reads were quality trimmed and mapped to the mouse genome (mm10) using Bowtie2. Uniquely mapped reads were filtered by removing alignments with a mapping quality of <10. ChIP–seq peaks were then retrieved using MACS2 (2.2.7.1) with the default parameters. Input samples were used as reference controls for background correction. From MACS2 output, ‘robust’ peaks were selected by specifying a minimum fold enrichment of 2 and restricting peak location to the promoter regions of genes. Bedtools software was used for peak subtraction. Peaks were annotated using the annotate. Peaks function on Homer (4.11). ChIP–PCR primers listed in Supplementary Table 7.

### SiRNA transfection

**Extended Data Fig. 10:**
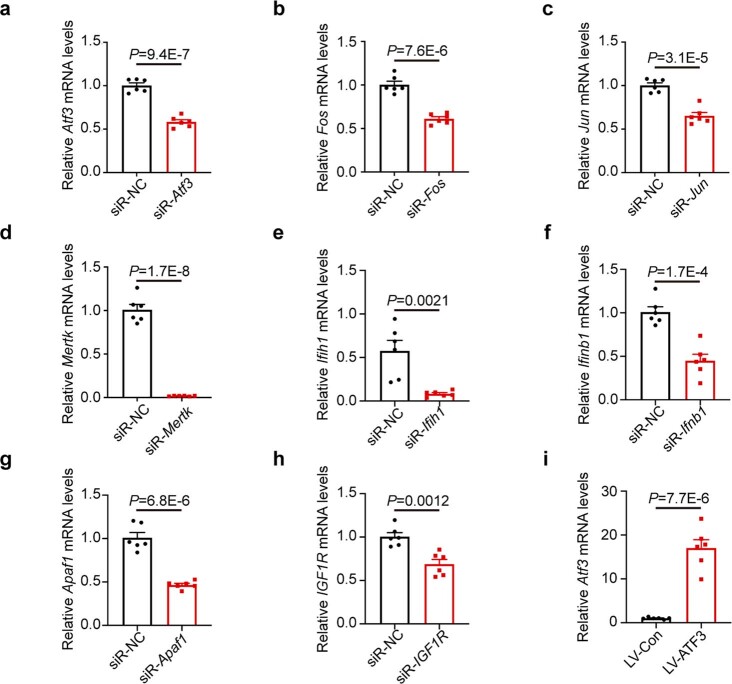
Efficiency of siRNA knockdown and lentiviral transfection. **a**–**h**. RT-qPCR of *Atf3* (**a**), *Fo*s (**b**), *Jun* (**c**), *Mertk* (**d**), *Ifih1* (**e**), *Ifnb1* (**f**), *Apaf1* (**g**) and *IGF1R* (**h**) knockdown efficiency in macrophages upon siRNA treatment (n = 6 biologically independent samples per group). **i**. RT-qPCR validation of *Atf3* overexpression efficiency by LV-ATF3 in macrophages (n = 6 biologically independent samples per group). Statistical significance was evaluated using two-tailed unpaired *t*-test (**a**–**i**). All data are presented as mean ± SEM. Source data

For gene-silencing studies, small interfering RNAs (siRNAs) against *Atf3*, *Fos*, *Jun*, *Ifih1*, *Ifnb1* and *Apaf1* were transfected into BMDMs; siRNA against IGF1R was transfected into HUVECs at a final concentration of 50 nM using Lipofectamine RNAiMAX transfection reagent (Invitrogen, catalog no. 13778150) according to the manufacturer’s recommendations^57^. The siRNA sequences are listed in Supplementary Table 8. The efficiency of knockdown was evaluated using RT–qPCR (Extended Data Fig. 10a–h).

### Lentivirus-mediated overexpression

ATF3 overexpression was achieved using lentiviral infection. Briefly, the lentiviral vector GV303 encoding mouse *Atf3* (LV-ATF3) and negative control (LV-Con) were constructed, packaged, purified and titrated at GeneChem Co. Ltd. For lentiviral-mediated gene transfer, LV-ATF3 or LV-Con was transfected into BMDM/GAS6-induced MerTK^+^ macrophages at a multiplicity of infection of 100 for 72 h. The overexpression efficiency of LV-ATF3 was evaluated using RT–qPCR (Extended Data Fig. 10i)^58^.

### ELISA

IGF1 levels were analyzed using a mouse IGF1 Quantikine ELISA Kit (R&D Systems, catalog no. MG100). GAS6 levels in mice were analyzed using a murine GAS6 ELISA kit (Cloud-Clone Corp., catalog no. SEA204Mu). Human plasma GAS6 levels were analyzed using a human GAS6 DuoSet ELISA kit (R&D Systems, catalog no. DY885B). All measurements were performed in duplicate.

### Human studies

#### Association of ATF3 SNPs with the risk of MACEs

In our study, we utilized the data collected at the UK Biobank (UKB) assessment centers at baseline, combined with information on incident disease events from the hospital and death registry; 39,707 individuals diagnosed as CAD (*International Classification of Diseases*, 10th edn (ICD-10) codes I20.0 and I21)^59^ at baseline were used in our study sample. The UKB study was approved by the North West Multi-Center Research Ethics Committee and all participants provided written informed consent to participate in the UKB study.

The MACEs were defined using inpatient hospital and death registry data linked to the UKB. MACEs included recurrent MI, heart failure and death because of cardiovascular disease (CVD). Recurrent MI was defined as ICD-10 code I22 and field ID 42000–42005. Heart failure was defined as ICD-10 code I50. Death because of CVD was defined using the same ICD-10 codes for different endpoints from the death registry. We used Plink 2.0 to analyze the association between the SNPs located in the region 100 kb upstream and downstream of ATF3 and MACEs in 39,707 individuals with CAD from UKB. The eQTLs of ATF3 were retrieved from public summary data of eQTLGen (https://www.eqtlgen.org), a consortium investigating the genetic architecture of blood gene expression, with a total of 31,684 individuals. Bubble plots were produced in R using ggplot2 package (v.3.3.2). Haploview software were used to perform linkage disequilibrium analysis.

#### Association of plasma GAS6 with the risk of MACEs

Patients with ACS were admitted to Anzhen Hospital of Capital Medical University (Beijing, China). We enrolled patients with ACS following a particular set of inclusion and exclusion criteria. The inclusion criteria were as follows: (1) patients (age ≥18 years) presenting within 5 d (preferably within 72 h) after pain onset with the main diagnosis of ST-elevation MI (STEMI) non-STEMI or unstable angina; and (2) known CAD, defined as status after MI, coronary artery bypass grafting (CABG), percutaneous coronary intervention or newly documented case of >50% stenosis of an epicardial coronary artery during the initial catheterization. The exclusion criteria encompassed patients with renal or hepatic dysfunction, a history of tumors and those receiving oral anticoagulation. Coronary angiography was performed using standard techniques. The diameter and angiographic flow of collateral vessels were semiquantitatively assessed using the Rentrop classification (class 0 = no visible filling of collaterals, class 1 = filling of side branches, class 2 = partial filling of the epicardial segment of the occluded vessel and class 3 = total filling of the epicardial segment). The degree of CCC was assessed using the Rentrop score and was classified as either poor (Rentrop 0 or 1) or good (Rentrop 2 or 3). Comparisons between good and poor CCC were performed within the overall cohort using an adjusted analysis with a 1:1 propensity score matching for age, sex, number of coronary lesions and left main CAD. Healthy controls consisted of age- and sex-matched subjects without cardiovascular risk factors and without a history of CAD, and were recruited from the health examination center in Anzhen Hospital. Information on demographic characteristics, history, clinical presentation, physical examination, imaging information and management was obtained from the medical records. All participants provided written informed consent. The present study was approved by the Institutional Review Board of Beijing Anzhen Hospital. The study design was conducted in accordance with the Declaration of Helsinki (ClinicalTrials, NCT03752515). Blood samples were collected at the time of hospital admission and they were drawn into coagulation-promoting tubes and centrifuged for 1 h at 2,000*g* for 10 min. All plasma samples were stored in aliquots at −80 °C until assayed.

After the initial hospitalization, the patients were followed up for a median of 343 d (interquartile range: 260–460 d). Annual contact was made via an initial telephone call to individuals and their relatives by a trained researcher. If any event was reported, the hospital was contacted for validation based on records and death certificates. We defined major adverse cardiovascular events as a composite of cardiac death, readmission for heart failure, recurrent unstable angina and repeat revascularization.

### Statistical analysis

Data are expressed as the mean ± s.e.m. and statistical analyses were performed using SPSS v.24.0 (IBM Corporation) and GraphPad Prism (v.8; GraphPad software). Normality was assessed using the Shapiro–Wilk test. If the data passed the normality evaluation, the unpaired, two-tailed Student’s *t*-test was used to compare two independent groups and a one-way (one variable) analysis of variance (ANOVA) or two-way (two variables) ANOVA followed by Tukey’s post hoc multiple-comparison test was used for comparisons between multiple groups. When data showed a non-normal distribution or a sample size <6, the Mann–Whitney *U*-test was used to compare two groups and the Kruskal–Wallis test for multiple groups, followed by Dunn’s multiple-comparison test, was used for post hoc comparisons. In all cases, *P* < 0.05 was considered statistically significant. For human studies, the *χ*² test or Fisher’s exact test was used for categorical variables. In Supplementary Table 5, the sample size was >40. In the 2 × 2 table data, the expected counts of all variables were >5 and we used the *χ*^2^ test. In the R × C table data, the expected counts of number of stenosed coronary vessels were >5 in all cells and we used the *χ*² test. Four cells (50%) of treatment had expected count <5, exceeding 1/5 and we used Fisher’s exact test. Continuous variables were compared between groups using either the Student’s *t*-test or the Mann–Whitney *U*-test. Logistic regression models, both multivariate/univariate, were used to calculate the odds ratios associated with plasma GAS6 levels and degree of CCC. We selected variables with significant differences (*P* < 0.05) between two groups of patients for adjustment and they included drinking, previous CABG and number of stenosed coronary vessels. The correlation was analyzed using Pearson’s correlation test. The distinguishing capacity of GAS6 was assessed using receiver operating curve analysis. Survival distributions were compared using the log(rank) test.

### Reporting summary

Further information on research design is available in the Nature Portfolio Reporting Summary linked to this article.

### Supplementary information

Reporting Summary

Supplementary Tables 1–8. Supplementary Table 1 Top 20 differentially expressed and predicted TFs in MerTK^+^ macrophage. Statistical significance was evaluated using two-tailed Wilcoxon’s rank-sum test. Supplementary Table 2 DEGs in rGAS6-stimulated BMDMs from ATF3-CKO and WT mice. Statistical significance was evaluated using a simple linear model and moderated Student’s *t*-statistics, implemented in the R package limma v.3.52.4. The corrected *P* values were calculated for multiple testing using the Benjamini–Hochberg FDR adjustment. Supplementary Table 3 DEG peaks between the LV-Con- and LV-ATF3-transfected macrophages from ChIP–seq. *P* values were calculated from the algorithm called bdgdiff in MACS2 software. Supplementary Table 4 SNPs at the intersection of MACE-associated SNPs and ATF3 eQTL variants. Statistical significance was evaluated using two-tailed Pearson’s correlations analysis. Supplementary Table 5 Baseline characteristics in patients with myocardial ischemia. Statistical significance was evaluated using two-tailed Mann–Whitney *U*-test (continuous variables) and *χ*^2^ test or Fisher’s exact test (categorical variables). Supplementary Table 6 DEGs of seven clusters of macrophages/monocytes. Statistical significance was evaluated using two-tailed Wilcoxon’s rank-sum test. Supplementary Table 7 A list of RT–qPCR primers and CHIP–PCR primers. Supplementary Table 8 A list of siRNA sequences.

### Source data

Source Data Fig. 1 Statistical source data.

Source Data Fig. 2 Statistical source data.

Source Data Fig. 3 Statistical source data.

Source Data Fig. 4 Statistical source data.

Source Data Fig. 5 Statistical source data.

Source Data Fig. 6 Statistical source data.

Source Data Fig. 7 Statistical source data.

Source Data Extended Data Fig. 1 Statistical source data.

Source Data Extended Data Fig. 2 Statistical source data.

Source Data Extended Data Fig. 2 Unprocessed western blots and gels.

Source Data Extended Data Fig. 3 Statistical source data.

Source Data Extended Data Fig. 4 Statistical source data.

Source Data Extended Data Fig. 5 Statistical source data.

Source Data Extended Data Fig. 6 Statistical source data.

Source Data Extended Data Fig. 7 Statistical source data.

Source Data Extended Data Fig. 8 Statistical source data.

Source Data Extended Data Fig. 9 Statistical source data.

Source Data Extended Data Fig. 10 Statistical source data.

## Data Availability

The microarrays reported in the present study have been deposited in the Gene Expression Omnibus under accession nos. GSE246338 and GSE246339, respectively, whereas the scRNA-seq raw data have been deposited in the same publicly available database under accession no. GSE247139. Other data are available in the main article and Supplementary Information. Source data are provided with this paper.

## Code availability

The analysis code in this paper is freely available on GitHub (https://github.com/CFTMlab/2023_Li_et_al_NCVR_ATF3_IR).
