# Supplementary material for: ATF3 coordinates the survival and proliferation of cardiac macrophages and protects against ischemia–reperfusion injury
Source: Nat Cardiovasc Res. 2024 Jan 4;3(1):28–45. doi: 10.1038/s44161-023-00392-x (PMC11358155; doi:10.1038/s44161-023-00392-x)
Supplement: Supplementary file 1 — Reporting Summary [file 44161_2023_392_MOESM1_ESM.pdf]

nature portfolio

Corresponding author(s): Yulin Li

Last updated by author(s): Oct 29, 2023

# Reporting Summary

Nature Portfolio wishes to improve the reproducibility of the work that we publish. This form provides structure for consistency and transparency in reporting. For further information on Nature Portfolio policies, see our Editorial Policies and the Editorial Policy Checklist.

## Statistics

For all statistical analyses, confirm that the following items are present in the figure legend, table legend, main text, or Methods section.

| n/a | Confirmed | |
|---|---|---|
| ☐ | ☒ | The exact sample size ($n$) for each experimental group/condition, given as a discrete number and unit of measurement |
| ☐ | ☒ | A statement on whether measurements were taken from distinct samples or whether the same sample was measured repeatedly |
| ☐ | ☒ | The statistical test(s) used AND whether they are one- or two-sided<br>*Only common tests should be described solely by name; describe more complex techniques in the Methods section.* |
| ☐ | ☒ | A description of all covariates tested |
| ☐ | ☒ | A description of any assumptions or corrections, such as tests of normality and adjustment for multiple comparisons |
| ☐ | ☒ | A full description of the statistical parameters including central tendency (e.g. means) or other basic estimates (e.g. regression coefficient) AND variation (e.g. standard deviation) or associated estimates of uncertainty (e.g. confidence intervals) |
| ☐ | ☒ | For null hypothesis testing, the test statistic (e.g. $F$, $t$, $r$) with confidence intervals, effect sizes, degrees of freedom and $P$ value noted<br>*Give P values as exact values whenever suitable.* |
| ☒ | ☐ | For Bayesian analysis, information on the choice of priors and Markov chain Monte Carlo settings |
| ☒ | ☐ | For hierarchical and complex designs, identification of the appropriate level for tests and full reporting of outcomes |
| ☐ | ☒ | Estimates of effect sizes (e.g. Cohen's $d$, Pearson's $r$), indicating how they were calculated |

*Our web collection on statistics for biologists contains articles on many of the points above.*

## Software and code

Policy information about availability of computer code

| | |
|---|---|
| Data collection | Single cell RNA sequencing libraries were generated using the 10× Chromium Single Cell 30 v2 reagent kit (10X Genomics). The resulting libraries were sequenced on an NovaSeq6000 platform (Illumina).<br>Flow cytometry data were acquired on a FACS Aria II flow cytometer (BD Biosciences).<br>Light microscopy images were captured on a Nikon Eclipse TE2000-S light microscope (Nikon).<br>Immunofluorescence images were taken with a Leica ST5 laser scanning confocal microscope (Leica).<br>RT-qPCR data were acquired on a iCycler iQ system (Bio-Rad).<br>Microarray data were sequenced using a Gene Chip Scanner 3000 7G (Affymetrix).<br>RNA-seq DNA libraries were sequenced on a HiSeq 2000 instrument (Illumina).<br>MRI was performed with a Bruker BioSpec70/20 small animal MRI system (Bruker).<br>microCT imaging was performed with a Siemens Inveon PET/CT scanner (Siemens).<br>Transthoracic echocardiographic was performed using a VisualSonics Vevo 2100 Ultrasound system (Visual Sonics). |

| Data analysis | scRNA-seq data were processed and analyzed with 10X CellRanger pipeline v2.1.2, R v4.2.1, Seurat v2.2.1, ggplot2 v3.3.6, stats v4.2.1, CellChat v1.5.0 and RCisTarget v1.16.0.<br>Flow cytometry data were analyzed with FACS Diva 6.1 software (BD Biosciences).<br>Light microscopy and immunofluorescence images were analyzed using ImageJ (Fiji) v2.1.0/1.53c software (NIH).<br>Microarray and RNA-seq data were processed and analyzed using R v4.2.1, limma v3.52.4, pheatmap v1.0.12, and clusterProfiler v4.4.4.<br>MRI images were analyzed using QMass MR v7.6 software (Medis).<br>3D reconstruction and quantification of the microCT datasets was performed using Inveon Research Workplace v2.2.0 software (Siemens).<br>Cardiac function were analyzed using Vevo Lab v3.2.0 software (Visual Sonics).<br>Statistical analysis was performed using SPSS version 24.0 (IBM Corporation) and GraphPad Prism (version 8). |
|---|---|

For manuscripts utilizing custom algorithms or software that are central to the research but not yet described in published literature, software must be made available to editors and reviewers. We strongly encourage code deposition in a community repository (e.g. GitHub). See the Nature Portfolio guidelines for submitting code & software for further information.

## Data

Policy information about availability of data

All manuscripts must include a data availability statement. This statement should provide the following information, where applicable:

- Accession codes, unique identifiers, or web links for publicly available datasets
- A description of any restrictions on data availability
- For clinical datasets or third party data, please ensure that the statement adheres to our policy

The microarrays reported in this paper have been deposited in the Gene Expression Omnibus under accession numbers GSE246338 and GSE246339, respectively, while the single-cell RNA-seq raw data have been deposited in the same publicly available database under accession number GSE247139. Other data are available in the main article and associated files. Source data are provided with this paper.

## Research involving human participants, their data, or biological material

Policy information about studies with human participants or human data. See also policy information about sex, gender (identity/presentation), and sexual orientation and race, ethnicity and racism.

| | |
|---|---|
| Reporting on sex and gender | N/A |
| Reporting on race, ethnicity, or other socially relevant groupings | N/A |
| Population characteristics | All participants were enrolled from Anzhen Hospital of Capital Medical University (Beijing, China). The degree of coronary collateral circulation (CCC) was assessed using the Rentrop score and was classified as either poor (Rentrop 0 or 1) or good (Rentrop 2 or 3). Comparisons between good and poor CCC were performed within the overall cohort using an adjusted analysis with a 1:1 propensity score matching for age, sex, number of coronary lesions, and left main coronary artery disease. |
| Recruitment | Patients with acute coronary syndrome (ACS) were admitted to Anzhen Hospital of Capital Medical University (Beijing, China). We enrolled patients with ACS following a particular set of inclusion and exclusion criteria. The inclusion criteria were as follows: i) Patients (age ≥ 18 years) presenting within 5 days (preferably within 72 h) after pain onset with the main diagnosis of ST-elevation myocardial infarction, non-ST-elevation myocardial infarction, or unstable angina; ii) known coronary artery disease, defined as status after myocardial infarction, coronary artery bypass grafting, percutaneous coronary intervention, or newly documented case of > 50% stenosis of an epicardial coronary artery during the initial catheterization. The exclusion criteria encompassed patients with renal or hepatic dysfunction, a history of tumor, and those receiving oral anticoagulation. |
| Ethics oversight | The study design and conduct complied with all relevant regulations regarding the use of human study participants and was conducted in accordance with the Declaration of Helsinki. (NCT03752515) |

Note that full information on the approval of the study protocol must also be provided in the manuscript.

# Field-specific reporting

Please select the one below that is the best fit for your research. If you are not sure, read the appropriate sections before making your selection.

☒ Life sciences ☐ Behavioural & social sciences ☐ Ecological, evolutionary & environmental sciences

For a reference copy of the document with all sections, see nature.com/documents/nr-reporting-summary-flat.pdf

# Life sciences study design

All studies must disclose on these points even when the disclosure is negative.

| | |
|---|---|
| Sample size | Sample size calculation was not performed. The sample size for animals experiments was determined based on common practice of the described experiments in the literature and our previous work (PMID: 30538339 and 31220942). The sample size for cohort was chosen based upon availability of patients meeting our study criteria for inclusion. |

| | |
|---|---|
| Data exclusions | No data was excluded from the analyses in our animals study. The clinical case exclusions are well described in the manuscript: The exclusion criteria encompassed patients with renal or hepatic dysfunction, a history of tumor, and those receiving oral anticoagulation. |
| Replication | Each sample is a biological replicate. All attempts at replication were successful. For scRNA-seq and microarray after cell sorting, cardiac cell suspensions from three hearts were pooled into a sample due to the small number of cells. There was no replication in cohort, because these were clinical observations. |
| Randomization | Animals of the same age, sex and genotype were randomly assigned to treatment groups. For in vitro and ex vivo experiments, cells and treatments were randomly assigned. For the cohort, there was no allocation to experimental or control groups in this study and so no randomisation. |
| Blinding | The investigators were blinded to IR/Sham operation of each animal and group allocation during data collection and analyses. |

# Reporting for specific materials, systems and methods

We require information from authors about some types of materials, experimental systems and methods used in many studies. Here, indicate whether each material, system or method listed is relevant to your study. If you are not sure if a list item applies to your research, read the appropriate section before selecting a response.

## Materials & experimental systems

| n/a | Involved in the study | |
|---|---|---|
| ☐ | ☒ | Antibodies |
| ☐ | ☒ | Eukaryotic cell lines |
| ☒ | ☐ | Palaeontology and archaeology |
| ☐ | ☒ | Animals and other organisms |
| ☐ | ☒ | Clinical data |
| ☒ | ☐ | Dual use research of concern |
| ☒ | ☐ | Plants |

## Methods

| n/a | Involved in the study | |
|---|---|---|
| ☐ | ☒ | ChIP-seq |
| ☐ | ☒ | Flow cytometry |
| ☒ | ☐ | MRI-based neuroimaging |

## Antibodies

Antibodies used

Flow Cytometry: PE-CF594 Rat Anti-Mouse CD45 (BD, 562420), BV510 Rat Anti-Mouse CD11b (BD, 562950), PE-Cy7 Rat Anti-Mouse F4/80 (eBioscience, 25-4801-82), PE-Cy7 Rat Anti-Mouse MerTK (BioLegend, 151522), BV650 Rat Anti-Mouse CCR2 (BioLegend, 150613), PE Rat Anti-Mouse LYVE1 (eBioscience, 12-0443-82), AF700 Rat Anti-Mouse MHC Class II (eBioscience, 56-5321-82), BV510 Mouse Anti-Mouse CX3CR1 (BioLegend, 149025), PE Rat Anti-Mouse CD31 (BD, 561073), APC Rat Anti-Mouse PDGFR-α (BD, 562777), PE Rat Anti-Mouse Ly6G (BD, 553128), FITC Rat Anti-Mouse CD3 (BioLegend, 100204), and APC-Cy7 Rat Anti-Mouse CD19 (BD, 561737) or isotype control antibody (BV510 IgG isotype control, BD, 562951, PE IgG isotype control, BD, 553930, and PE-Cy7 IgG isotype control, BD, 552784)

Immunofluorescence: Rabbit anti-MerTK (Abcam, ab300136), Rat anti-F4/80 (Abcam, ab6640), Rabbit anti-Ki67 (Abcam, ab15580), Rabbit anti-ATF3 (Abcam, ab207434), anti-α-actinin (Sigma, A7732), Rabbit anti-CD31 (Abcam, ab222783), Rabbit anti-Col1A2 (Abcam, ab208638), Rabbit anti-TREM2 (Invitrogen, PA5-119690), Rabbit anti-LYVE1 (Invitrogen, MA5-32512), Rabbit anti-CD74 (Abcam, ab289885), Rabbit anti-CX3CR1 (Abcam, ab308613), Rabbit anti-IFIH1 (Abcam, ab79055), Rabbit anti-IFNB1 (Invitrogen, PA5-102429), Rabbit and anti-APAF1 (Invitrogen, MA5-32082), Goat anti-Mouse Alexa Fluor 488 Secondary Antibody (Invitrogen, A-11001), Goat anti-Rabbit Alexa Fluor 488 Secondary Antibody (Invitrogen, A-11008), Goat anti-Rat FITC Secondary Antibody (Invitrogen, A-11006), Goat anti-Mouse Alexa Fluor 555 Secondary Antibody (Invitrogen, A-21422), Donkey anti-Rabbit Alexa Fluor 555 Secondary Antibody (Invitrogen, A-31572), Goat anti-Rabbit Alexa Fluor 633 (Invitrogen, A-21070)

WB: Rabbit anti-phospho-Akt (Cell Signaling Technology, #13038), Rabbit anti-Akt (Cell Signaling Technology, #4691), Rabbit anti-phospho-MERTK (Abcam, ab192649; 1:1000), Rabbit anti-MERTK (Abcam, ab300136; 1:1000), Rabbit anti-ATF3 (Abcam, ab207434; 1:1000), Mouse anti-GAPDH (ZSGB-BIO, TA08), Anti-Mouse IgG Antibody DyLight™800 (Rockland Immunochemicals, 610-145-002), Anti-Rabbit IgG Antibody DyLight™800 (Rockland Immunochemicals, 611-145-002)

Validation

PE-CF594 Rat Anti-Mouse CD45 (https://www.bdbiosciences.com/zh-cn/products/reagents/flow-cytometry-reagents/research-reagents/single-color-antibodies-ruo/pe-cf594-rat-anti-mouse-cd45.562420),
BV510 Rat Anti-Mouse CD11b (https://www.bdbiosciences.com/zh-cn/products/reagents/flow-cytometry-reagents/research-reagents/single-color-antibodies-ruo/bv510-rat-anti-cd11b.562950),
PE-Cy7 Rat Anti-Mouse F4/80 (https://www.thermofisher.cn/cn/zh/antibody/product/F4-80-Antibody-clone-BM8-Monoclonal/25-4801-82),
PE-Cy7 Rat Anti-Mouse MerTK (https://www.biolegend.com/en-us/products/pecyanine7-anti-mouse-mertk-mer-antibody-19635),
BV650 Rat Anti-Mouse CCR2 (https://www.biolegend.com/en-us/products/brilliant-violet-650-anti-mouse-cd192-ccr2-antibody-15387),
PE Rat Anti-Mouse LYVE1 (https://www.thermofisher.cn/cn/zh/antibody/product/LYVE1-Antibody-clone-ALY7-Monoclonal/12-0443-82),
AF700 Rat Anti-Mouse MHC Class II (https://www.thermofisher.cn/cn/zh/antibody/product/MHC-Class-II-I-A-I-E-Antibody-clone-M5-114-15-2-Monoclonal/56-5321-82),
BV510 Mouse Anti-Mouse CX3CR1 (https://www.biolegend.com/en-us/products/brilliant-violet-510-anti-mouse-cx3cr1-antibody-11853)

PE Rat Anti-Mouse CD31 (https://www.bdbiosciences.com/zh-cn/products/reagents/flow-cytometry-reagents/research-reagents/single-color-antibodies-ruo/pe-rat-anti-mouse-cd31.561073),
APC Rat Anti-Mouse PDGFR-α (https://www.bdbiosciences.com/zh-cn/products/reagents/flow-cytometry-reagents/research-reagents/single-color-antibodies-ruo/apc-rat-anti-mouse-cd140a.562777),
PE Rat Anti-Mouse Ly6G (https://www.bdbiosciences.com/zh-cn/products/reagents/flow-cytometry-reagents/research-reagents/single-color-antibodies-ruo/pe-rat-anti-mouse-ly-6g-and-ly-6c.553128),
FITC Rat Anti-Mouse CD3 (https://www.biolegend.com/en-us/products/fitc-anti-mouse-cd3-antibody-45),
APC-Cy7 Rat Anti-Mouse CD19 (https://www.bdbiosciences.com/zh-cn/products/reagents/flow-cytometry-reagents/research-reagents/single-color-antibodies-ruo/apc-cy-7-rat-anti-mouse-cd19.561737),
BV510 IgG isotype control (https://www.bdbiosciences.com/zh-cn/products/reagents/flow-cytometry-reagents/research-reagents/flow-cytometry-controls-and-lysates/bv510-rat-igg2b-isotype-control.562951)
PE IgG isotype control (https://www.bdbiosciences.com/zh-cn/products/reagents/flow-cytometry-reagents/research-reagents/flow-cytometry-controls-and-lysates/pe-rat-igg2a-isotype-control.553930)
PE-Cy7 IgG isotype control (https://www.bdbiosciences.com/zh-cn/products/reagents/flow-cytometry-reagents/research-reagents/flow-cytometry-controls-and-lysates/pe-cy-7-rat-igg2a-isotype-control.552784)
Rabbit anti-MerTK (https://www.abcam.cn/mertk-antibody-epr26359-12-ab300136.html),
Rat anti-F4/80 (https://www.abcam.cn/f480-antibody-cia3-1-macrophage-marker-ab6640.html),
Rabbit anti-Ki67 (https://www.abcam.cn/ki67-antibody-ab15580.html),
Rabbit anti-ATF3 (https://www.abcam.cn/atf3-antibody-epr19488-chip-grade-ab207434.html),
Mouse anti-α-actinin (https://www.sigmaaldrich.cn/CN/zh/product/sigma/a7732),
Rabbit anti-CD31 (https://www.abcam.cn/cd31-antibody-epr17260-263-ab222783.html),
Rabbit anti-Col1A2 (https://www.abcam.cn/col1a2-antibody-ab208638.html),
Rabbit anti-TREM2 (https://www.thermofisher.cn/cn/zh/antibody/product/TREM2-Antibody-Polyclonal/PA5-119690),
Rabbit anti-LYVE1 (https://www.thermofisher.cn/cn/zh/antibody/product/LYVE1-Antibody-clone-JF0979-Recombinant-Monoclonal/MA5-32512),
Rabbit anti-CD74 (https://www.abcam.cn/products/primary-antibodies/cd74-antibody-epr25399-94-ab289885.html),
Rabbit anti-CX3CR1 (https://www.abcam.cn/products/primary-antibodies/cx3cr1-antibody-epr24267-2-ab308613.html),
Rabbit anti-IFIH1 (https://www.abcam.cn/products/primary-antibodies/mda5-antibody-ab79055.html),
Rabbit anti-IFNB1 (https://www.thermofisher.cn/cn/zh/antibody/product/IFN-beta-Antibody-Polyclonal/PA5-102429),
Rabbit anti-APAF1 (https://www.thermofisher.cn/cn/zh/antibody/product/APAF1-Antibody-clone-SY22-02-Recombinant-Monoclonal/MA5-32082)
Goat anti-Mouse Alexa Fluor 488 Secondary Antibody (https://www.thermofisher.cn/cn/zh/antibody/product/Goat-anti-Mouse-IgG-H-L-Cross-Adsorbed-Secondary-Antibody-Polyclonal/A-11001),
Goat anti-Rabbit Alexa Fluor 488 Secondary Antibody (https://www.thermofisher.cn/cn/zh/antibody/product/Goat-anti-Rabbit-IgG-H-L-Cross-Adsorbed-Secondary-Antibody-Polyclonal/A-11008),
Goat anti-Rat FITC Secondary Antibody (https://www.thermofisher.cn/cn/zh/antibody/product/Goat-anti-Rat-IgG-H-L-Cross-Adsorbed-Secondary-Antibody-Polyclonal/A-11006),
Goat anti-Mouse Alexa Fluor 555 Secondary Antibody (https://www.thermofisher.cn/cn/zh/antibody/product/Goat-anti-Mouse-IgG-H-L-Cross-Adsorbed-Secondary-Antibody-Polyclonal/A-21422),
Donkey anti-Rabbit Alexa Fluor 555 Secondary Antibody (https://www.thermofisher.cn/cn/zh/antibody/product/Donkey-anti-Rabbit-IgG-H-L-Highly-Cross-Adsorbed-Secondary-Antibody-Polyclonal/A-31572),
Goat anti-Rabbit Alexa Fluor 633 Secondary Antibody (https://www.thermofisher.cn/cn/zh/antibody/product/Goat-anti-Rabbit-IgG-H-L-Cross-Adsorbed-Secondary-Antibody-Polyclonal/A-21070)
Rabbit anti-phospho-Akt (https://www.cellsignal.cn/products/primary-antibodies/phospho-akt-thr308-d25e6-xp-rabbit-mab/13038?site-search-type=Products&N=4294956287&Ntt=13038&fromPage=plp&_requestid=795628),
Rabbit anti-Akt (https://www.cellsignal.cn/products/primary-antibodies/akt-pan-c67e7-rabbit-mab/4691?site-search-type=Products&N=4294956287&Ntt=4691&fromPage=plp&_requestid=795788),
Rabbit anti-phospho-MERTK (https://www.abcam.cn/products/primary-antibodies/mertk-phospho-y749--tyro3-phospho-y681-antibody-ab192649.html),
Anti-Mouse IgG Antibody DyLight™800 (https://www.rockland.com/categories/secondary-antibodies/mouse-igg-hl-antibody-dylight-800-conjugated-610-145-002/),
Anti-Rabbit IgG Antibody DyLight™800 (https://www.rockland.com/categories/secondary-antibodies/rabbit-igg-hl-antibody-dylight-800-conjugated-611-145-002/)

## Eukaryotic cell lines

Policy information about cell lines and Sex and Gender in Research

| | |
|---|---|
| Cell line source(s) | HUVECs (ATCC, CRL-1730) |
| Authentication | The cell line was purchased and authenticated by the commercial provider (ATCC). |
| Mycoplasma contamination | The cell providers guaranteed cell lines are free from mycoplasma contamination. No further testing was performed here. |
| Commonly misidentified lines (See ICLAC register) | None |

## Animals and other research organisms

Policy information about studies involving animals; ARRIVE guidelines recommended for reporting animal research, and Sex and Gender in Research

| | |
|---|---|
| Laboratory animals | Conditional deletion of ATF3 in myeloid lineages (ATF3-CKO) or resident cardiac macrophages was achieved by crossing ATF3flox/flox mice with Lyz2-Cre transgenic mice or Cx3cr1-Cre transgenic mice and breeding them to homozygosity. Cre- littermate mice served |

as the control. All experimental animals were maintained on a C57BL/6 genetic background. ATF3flox/flox mice were generated by Cyagen Biosciences, Inc. (Suzhou, China). Genotyping primers for ATF3flox/flox were as follows: F1, 5’-TTAGTTTGGAAGTGGATGGTGCATG-3’; R1, 5’-CGCCCTTGCTCACCATCTATAAAAT-3’; and R2, 5’-CTTGGAACAACTTTACCCATCCCC-3’. Lyz2-Cre (Stock No: 004781) and Cx3cr1-Cre mice (Stock No: 025524) were purchased from Jackson Laboratory. Male C57BL/6 mice were purchased from Viewsolid Biotech (Stock No: VSM10001, Beijing, China). Ten- to twelve-week-old male mice were used in the experiments. All animals were housed under pathogen-free conditions and allowed ad libitum access to food and water. The room was maintained under controlled temperature (20–25°C), humidity (30–70%), and light exposure cycle (12-h light and dark cycle) conditions.

| | |
|---|---|
| Wild animals | No wild animals were used in the study. |
| Reporting on sex | Female sex hormones may be beneficial in protecting the heart. IR surgery causes a mild and variable phenotype in females. Thus, only male mice were used in this study. |
| Field-collected samples | No field collected samples were used in the study. |
| Ethics oversight | All experiments involving animals were conducted based on the Guidelines on the Use and Care of Laboratory Animals and approved by the Animal Subjects Committee of Beijing Anzhen Hospital, Capital Medical University. |

Note that full information on the approval of the study protocol must also be provided in the manuscript.

# Clinical data

Policy information about clinical studies
All manuscripts should comply with the ICMJE guidelines for publication of clinical research and a completed CONSORT checklist must be included with all submissions.

| | |
|---|---|
| Clinical trial registration | NCT03752515 |
| Study protocol | The full trail protocol can be accessed at "Human study" in the manuscript. |
| Data collection | Information on demographic characteristics, history, clinical presentation, physical examination, imaging information, and management was obtained from the medical records. Blood samples were collected at the time of hospital admission, and they were drawn into coagulation-promoting tubes and centrifuged for 1 h at 2000 × g for 10 min. |
| Outcomes | We defined major adverse cardiovascular events as a composite of cardiac death, readmission for heart failure, recurrent unstable angina, and repeat revascularization. |

# Plants

| | |
|---|---|
| Seed stocks | N/A |
| Novel plant genotypes | N/A |
| Authentication | N/A |

# ChIP-seq

## Data deposition

☐ Confirm that both raw and final processed data have been deposited in a public database such as GEO.

☐ Confirm that you have deposited or provided access to graph files (e.g. BED files) for the called peaks.

| | |
|---|---|
| Data access links<br>*May remain private before publication.* | ChIP-seq processed data are available in the supplementary materials (Tables S3). |
| Files in database submission | *Provide a list of all files available in the database submission.* |
| Genome browser session<br>(e.g. UCSC) | *Provide a link to an anonymized genome browser session for "Initial submission" and "Revised version" documents only, to enable peer review. Write "no longer applicable" for "Final submission" documents.* |

## Methodology

| | |
|---|---|
| Replicates | None |
| Sequencing depth | ATF3, ~10,-20 million paired-end reads were sequenced and ~8-16 million paired-end reads were uniquely mapped. The read length is 150 bp.<br>IgG, ~12,-22 million paired-end reads were sequenced and ~10-20- million paired-end reads were uniquely mapped. The read length is 150 bp. |

| | |
|---|---|
| Antibodies | Rabbit anti-ATF3 antibody (Abcam, ab207434) ,<br>Rabbit IgG (Abcam, ab172730) |
| Peak calling parameters | Mapping:<br>MACS2 (version 2.1.0) was used for peak calling with the following parameters: Pvalue = 0.01, extsize = 800 using IgG as input controls. Peak summits were extended by 3000 bp on either side for merging and downstream analysis.<br>ChIP-Seq:<br>Filtered bam files were used for peak calling by MACS2 for ATF3 (-log10Pvalue > 2 and FC > 2). |
| Data quality | For ChIP-sequencing, the concentration and quality of the DNA fragments were assessed on Qubit Fluorometer and Agilent Bioanalyzer 2100. DNA was end-repaired, followed by the addition of an A base to the 3′ ends, adaptor ligation, and finally PCR amplification. Ligation products were size-selected (175–225 bp) and assessed using Agilent Bioanalyzer. The libraries were pooled and sequenced using the Illumina HiSeq2000 platform. Reads were quality-trimmed and mapped to the mouse genome (mm10) using Bowtie2. Uniquely mapped reads were filtered by removing alignments with a mapping quality of less than 10. |
| Software | Bowtie2 was used to mapping reads to mouse genome.<br>Samtools was used to extract uniquely mapped reads, and remove duplicated reads.<br>Bedtools was used for determining read coverage within peaks.<br>MACS2, ChIPseeker and Homer were used for peak calling, peak annotation and motif search.<br>Integrative Genome Viewer was used for visualizing peaks and data analysis. |

# Flow Cytometry

## Plots

Confirm that:

☒ The axis labels state the marker and fluorochrome used (e.g. CD4-FITC).

☒ The axis scales are clearly visible. Include numbers along axes only for bottom left plot of group (a 'group' is an analysis of identical markers).

☒ All plots are contour plots with outliers or pseudocolor plots.

☒ A numerical value for number of cells or percentage (with statistics) is provided.

## Methodology

| | |
|---|---|
| Sample preparation | Hearts were perfused with 15 mL of pre-chilled saline containing 25 U/mL heparin sodium salt (Sigma Aldrich, H3149) for 5 min and then collected following IR. Left ventricle tissues were isolated, minced with fine scissors, and subjected to an enzymatic digestion solution containing collagenase II (200 U/mL, ThermoFisher, 17101015) and dispase II (1 U/mL, Roche, 04942078001) at 37°C for 30 min. Cells were collected and filtered (40 μm) to generate a single-cell suspension. The suspensions were washed with PBS containing 0.04% BSA. The cells were then incubated with fluorescently labeled anti-mouse antibodies, namely, PE-CF594-CD45 (BD, 562420), BV510-CD11b (BD, 562950), PE-Cy7-F4/80 (eBioscience, 25-4801-82), PE-F4/80 (BD, 565410), PE-Cy7-MerTK (BioLegend, 151522), BV650-CCR2 (BioLegend, 150613), PE-LYVE1 (eBioscience, 12-0443-82), AF700-MHC Class II (eBioscience, 56-5321-82), BV510-CX3CR1 (BioLegend, 149025), PE-CD31 (BD, 561073), APC-PDGFR-α (BD, 562777), PE-Ly6G (BD, 553128), FITC-CD3 (BioLegend, 100204), and APC-Cy7-CD19 (BD, 561737) or isotype control antibody (BV510 IgG isotype control, BD, 562951, PE IgG isotype control, BD, 553930, and PE-Cy7 IgG isotype control, BD, 552784) at 4°C for 40 min. |
| Instrument | BD FACS Aria II flow cytometer (Becton Dickinson) |
| Software | FACS Diva 6.1 software (BD Biosciences) |
| Cell population abundance | Purity of sorted cells was >95% as assessed by confirmatory flow cytometry. |
| Gating strategy | All cell populations were pregated on viable and single cells.<br>MerTK+Mϕ: CD45+CD11b+MerTK+CCR2-;<br>LYVE1+Mϕ: CD45+CD11b+MerTK+CCR2-LYVE1+;<br>MHCII+Mϕ: CD45+CD11b+MerTK+CCR2-LYVE1-MHCII+;<br>TREM2+Mϕ: CD45+CD11b+MerTK+CCR2-LYVE1-MHCII-TREM2+;<br>Annexin V+MerTK+Mϕ: CD45+CD11b+MerTK+CCR2-Annexin V+;<br>EdU+MerTK+Mϕ: CD45+CD11b+MerTK+CCR2-EdU+;<br>Neutrophil: CD45+LY6G+;<br>T cell: CD45+LY6G-CD3+;<br>B cell: CD45+LY6G-CD3-CD19+;<br>Endothelial cell: CD45-PDGFRα-CD31+;<br>Mϕ: CD45+CD11b+F4/80+;<br>Fibroblast:  CD45-CD11b-CD31-PDGFR-α+;<br>Cardiomyocytes: CD45-CD11b-CD31-PDGFR-α-;<br>CX3CR1-Mϕ: CD45+CD11b+F4/80+CX3CR1-;<br>CX3CR1+Mϕ: CD45+CD11b+F4/80+CX3CR1+; |

☒ Tick this box to confirm that a figure exemplifying the gating strategy is provided in the Supplementary Information.
