# Supplementary figures and images for: ATF3 coordinates the survival and proliferation of cardiac macrophages and protects against ischemia–reperfusion injury
Source: Nat Cardiovasc Res. 2024 Jan 4;3(1):28–45. doi: 10.1038/s44161-023-00392-x (PMC11358155; doi:10.1038/s44161-023-00392-x)

Uncropped blots

Extended Data Fig. 2g

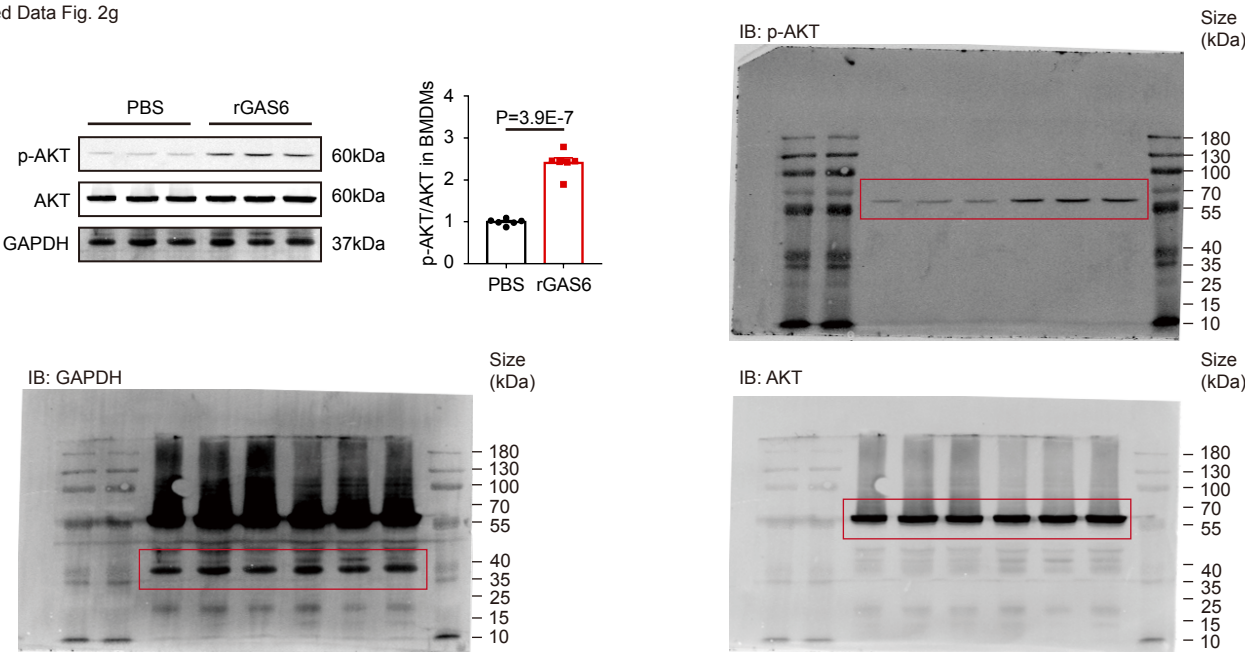

Extended Data Fig. 2k, l

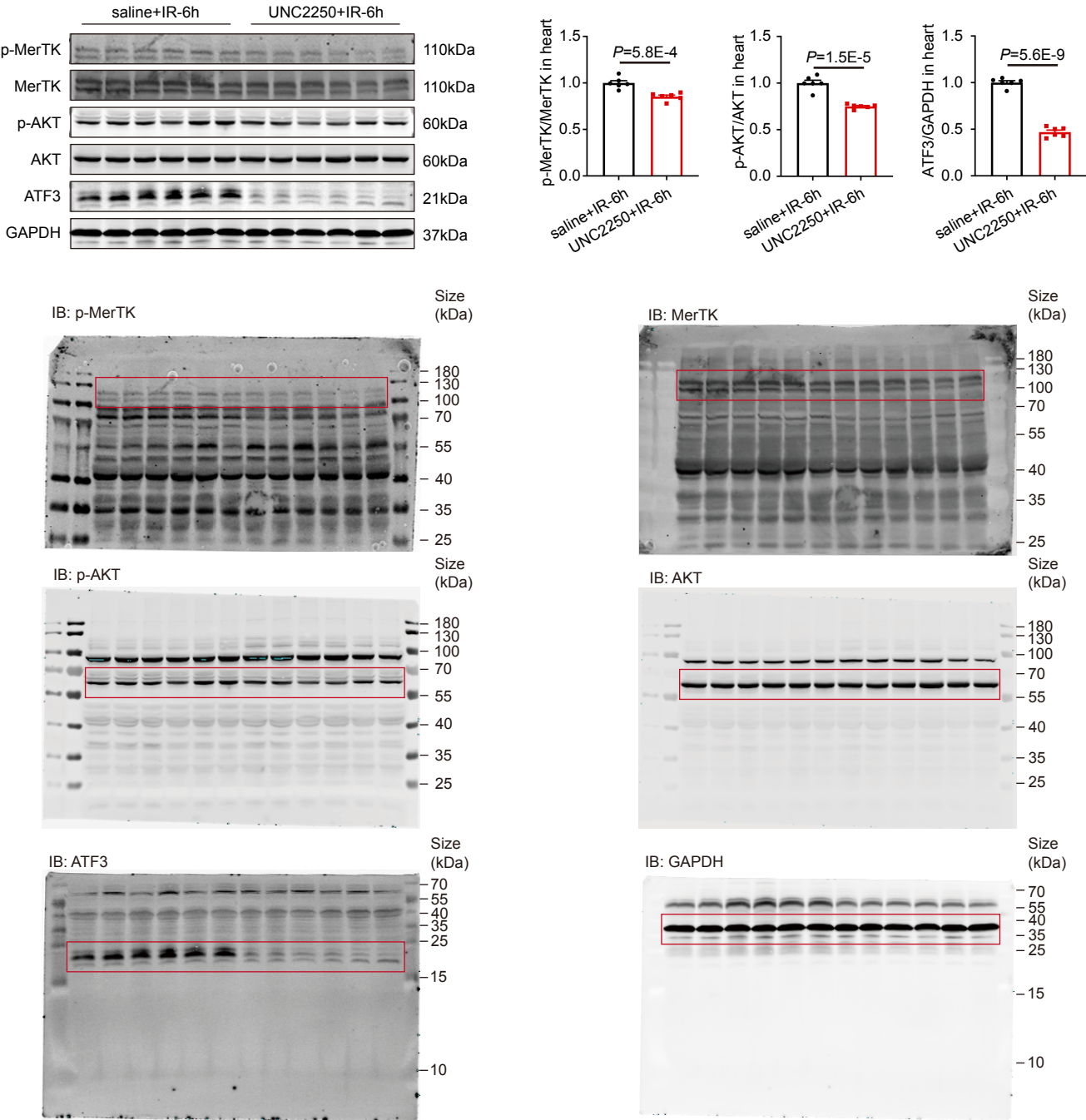

Supplement: Supplementary file 12 — Unprocessed western blots and gels. [file 44161_2023_392_MOESM12_ESM.pdf]
